# Functional Coupling and Longitudinal Outcome Prediction in First-Episode Psychosis

**DOI:** 10.1016/j.bpsgos.2025.100589

**Published:** 2025-08-11

**Authors:** Isaac Z. Pope, Sidhant Chopra, Alexander Holmes, Shona M. Francey, Brian O’Donoghue, Vanessa L. Cropley, Barnaby Nelson, Hok Pan Yuen, Kelly Allott, Mario Alvarez-Jimenez, Susy Harrigan, Christos Pantelis, Andrew Thompson, Stephen J. Wood, Patrick D. McGorry, Alex Fornito

**Affiliations:** a Turner Institute for Brain and Mental Health, School of Psychological Science, Monash University, Clayton, Victoria, Australia; b Monash Biomedical Imaging, Monash University, Clayton, Victoria, Australia; c Orygen, Parkville, Victoria, Australia; d Centre for Youth Mental Health, The University of Melbourne, Melbourne, Victoria, Australia; e Oxford University Centre for Integrative Neuroimaging, University of Oxford, Oxford, United Kingdom; f St. Vincent’s University Hospital, Dublin, Ireland; g School of Medicine, University College Dublin, Dublin, Ireland; h Melbourne Neuropsychiatry Centre, Department of Psychiatry, University of Melbourne, Melbourne, Victoria, Australia; i Centre for Mental Health, Melbourne School of Global and Population Health, The University of Melbourne, Parkville, Victoria, Australia; j Western Hospital Sunshine, St. Albans, Victoria, Australia; k School of Psychology, University of Birmingham, Edgbaston, United Kingdom

**Keywords:** Connectome-based predictive modeling, First-episode psychosis, Functional coupling, Longitudinal prediction, Meta-matching, Ridge regression

## Abstract

**Background:**

Clinical outcomes following the first episode of psychosis (FEP) are highly heterogeneous across patients. The identification of prognostic biomarkers would greatly facilitate personalized treatments. Patients with psychosis often display brainwide disruptions of interregional functional coupling (FC), with some being linked to symptom severity and remission. Thus, FC may have prognostic potential for people experiencing psychosis.

**Methods:**

Fifty-five antipsychotic-naïve patients with FEP (51% female, ages 15–25 years) were randomized to receive either antipsychotic or placebo tablets for 6 months alongside psychosocial interventions. Functional magnetic resonance imaging was conducted at baseline and after 3 months to evaluate whether baseline FC or 3-month change in FC could predict 6- and 12-month changes in symptoms and functioning, quantified using the Brief Psychiatric Rating Scale and the Social and Occupational Functioning Assessment Scale, respectively. We considered 3 different cross-validated prediction algorithms: 1) connectome-based predictive modeling, 2) kernel ridge regression, and 3) multilayer meta-matching. Each prediction model comprised 35 to 49 individuals.

**Results:**

All models showed poor performance in predicting patients’ 6- and 12-month changes in symptoms and functioning (all *r_mean_* < 0.3), and no model achieved significance via permutation testing (all *p* > .05).

**Conclusions:**

Our findings suggest that brainwide measures of FC may not be suitable for predicting extended clinical outcomes over a 6- to 12-month period in patients with FEP.

## Plain Language Summary

Clinical outcomes after the first episode of psychosis vary greatly between individuals. Developing personalized treatments requires first identifying reliable predictors of outcomes. We used three algorithms to evaluate whether functional coupling between brain regions could predict the outcomes of patients with first-episode psychosis following 6 months of psychosocial intervention and either antipsychotic or placebo treatment. All algorithms failed to accurately predict patients’ 6- and 12-month changes in symptoms and functioning, suggesting that brain-wide functional coupling may not be useful for guiding personalized treatment.

Clinical outcomes following the first episode of psychosis (FEP) are heterogeneous in both symptoms and functioning, ranging from permanent recovery to chronic and severe illness (1, 2, 3, 4). While early intervention with pharmacological and psychosocial treatments has been shown to improve clinical outcomes in patients with FEP (5, 6, 7, 8, 9, 10), the efficacy and tolerability of these treatments vary considerably between individuals (11,12). Moreover, some patients with FEP may recover through psychosocial treatment alone (13, 14, 15, 16). Current clinical guidelines for FEP address this heterogeneity via trial and error in prescription (17,18), potentially delaying remission and recovery (19). Therefore, the identification of reliable predictors of patients’ clinical outcomes is a necessary step toward the development of personalized treatment strategies.

Across the psychosis spectrum, magnetic resonance imaging (MRI) studies have revealed disruptions in both the structural connectivity between different brain regions (20, 21, 22) and the patterns of interregional functional coupling (FC) (i.e., correlated activity) that these connections support (21,23, 24, 25, 26, 27, 28, 29, 30, 31). Some of these FC disruptions are apparent at the onset of psychosis (23, 24, 25,30,31) and in high-risk individuals (32), and are related to different symptoms (32, 33, 34, 35) [but also see (36)]. Most importantly, prospective studies have found associations between patients’ baseline FC patterns and changes in their symptoms and functioning following treatment (31,37, 38, 39, 40, 41, 42). Despite the promise of this work, the analyses have generally relied on within-sample quantification of associations between FC and outcomes, which may inflate effect size estimates due to overfitting (43). Therefore, a thorough assessment of the generalizability of any putative prognostic biomarker requires analytic strategies such as cross-validation (43).

Some studies have reported cross-validated evidence that baseline FC can predict longitudinal symptom changes in patients with FEP (44, 45, 46, 47, 48, 49, 50), but most only involved short-term outcomes (i.e., up to 16 weeks postbaseline) in patients diagnosed with schizophrenia, potentially impeding the generalizability of their findings to the kinds of transdiagnostic FEP cohorts encountered in real-world clinical care (51). Moreover, the focus on predicting symptom changes may not translate into an impact on functional outcomes, because the two are not always related (52,53). Functional recovery in domains such as social relationships, educational attainment, and occupational functioning is particularly important in this regard, as it may address patient needs better than symptom recovery (54). Critically, all previous studies only used a single prediction algorithm rather than comparing the performance of multiple gold-standard approaches, which limits our ability to establish reliable and reproducible prediction methods for FEP.

In this study, we applied multiple cross-validated prediction algorithms to task-free resting-state functional MRI (fMRI) and clinical outcome data from STAGES (Staged Treatment and Acceptability Guidelines in Early Psychosis Study), a triple-blind randomized control trial of antipsychotics in previously antipsychotic-naïve patients with FEP (13,55). Our aim was to assess whether patients’ baseline resting-state FC could predict changes in their symptoms and functioning after 6 and 12 months. Because our previous analysis of this sample revealed associations between some 3-month changes in FC (ΔFC) and 12-month changes in symptoms and functioning (22), we also evaluated whether ΔFC could predict the same outcomes.

## Methods and Materials

### Design and Participants

**Figure 1:**
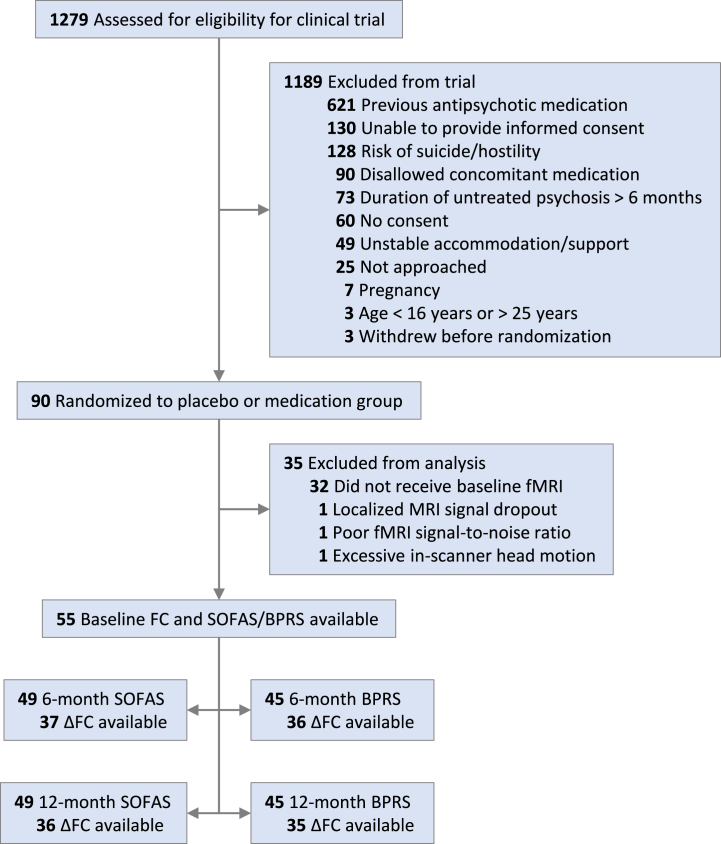
Flow diagram for patients involved in the larger clinical trial and in the current analysis. Patients without 3-month change in functional coupling (ΔFC) data available either discontinued the trial before the 3-month scan, refused the scan, or had localized signal dropout. The bottom 4 boxes indicate sample sizes used for the different prediction models. BPRS, Brief Psychiatric Rating Scale; fMRI, functional magnetic resonance imaging; SOFAS, Social and Occupational Functioning Assessment Scale.

This study used clinical and neuroimaging data from STAGES (13,55), which was approved by the Melbourne Health Human Research and Ethics Committee and registered under the Australian New Zealand Clinical Trials Registry (ACTRN12607000608460) in November 2007. Ninety patients with FEP (ages 15–25 years) were recruited between 2008 and 2016 at the Early Psychosis Prevention and Intervention Centre at Orygen Youth Health in Melbourne, Australia (Figure 1). Eligibility requirements comprised 1) meeting criteria for a psychotic disorder in DSM-IV via a structured clinical interview; 2) a duration of untreated psychosis <6 months; 3) ability to provide informed consent; 4) low risk of suicidality, self-harm, or hostility; 5) negligible lifetime use of antipsychotics or present use of mood stabilizers; 6) stable housing and support; and 7) no pregnancy. We used a transdiagnostic approach because diagnoses are often unstable in early psychosis (56, 57, 58, 59, 60, 61, 62, 63), treatment options are similar across diagnoses (17,18), diagnostic constructs may not accurately delineate subgroups of patients with FEP (64,65), and changes in symptoms and functioning provide the most clinically salient information. Patients were randomized to receive antipsychotic medication (risperidone or paliperidone) or matched placebo tablets for 6 months, together with psychosocial intervention in the form of cognitive behavioral case management (66). Further details about the trial design, including safety protocols, can be found in the Supplement and previous publications (13,55).

Patients’ functioning and symptoms were measured at baseline and after 6 and 12 months via the Social and Occupational Functioning Assessment Scale (SOFAS) and the Brief Psychiatric Rating Scale (BPRS) (67), respectively. Patients’ total scores on these inventories were preregistered as the primary and secondary outcomes of the larger clinical trial, respectively. Because we have previously demonstrated no differences in these outcomes between the placebo and medicated patients at 6 or 12 months (13), the treatment groups were combined in predictive models to maximize statistical power and generalizability. Because dichotomizing of patients into responders and nonresponders is statistically inefficient in ignoring within-group variability (68) and is not supported by a consensus definition of clinical and functional recovery (3), we defined patients’ clinical outcomes as continuous proportional change scores (*y*_2_ − *y*_1_) /*y*_1_, where *y*_1_ and *y*_2_ denote their SOFAS or BPRS score at baseline and at a later time point (6 or 12 months), respectively. Despite attrition throughout the trial, most patients who discontinued their participation in the trial still chose to complete clinical assessments. We included their data in our analysis to maximize statistical power and generalizability. For example, the 6-month predictive models included placebo-arm patients who commenced medication due to symptom exacerbation and received no 3-month scan but nevertheless completed 6-month assessments. No data imputation was performed.

### MRI Acquisition and Processing

A 3T Siemens Trio Tim scanner with a 32-channel head coil was used to acquire T1-weighted anatomical MRI and resting-state fMRI data at the Royal Children’s Hospital in Melbourne, Australia. Of the 90 patients in the clinical trial, 58 and 39 were scanned at baseline and after 3 months, respectively. We used the software MRIQC to automatically compute image quality metrics for each scan (69) and then excluded 2 baseline scans and one 3-month scan from further analysis due to localized signal dropout or high noise. Because ΔFC cannot be calculated from a single scan, the 3-month counterpart to one of the baseline scans was similarly excluded. We removed 1 scan due to excessive head motion, classified using standard exclusion criteria (70). The remaining scans were preprocessed via fMRIPrep version 1.4.1 (71) and then denoised using a pipeline previously shown to mitigate the impacts of noise sources such as head motion and non-neuronal physiological fluctuations (70). For each voxel in a scan, this involved linear detrending; regressing out motion-related signals identified by ICA-AROMA (Independent Component Analysis - Automatic Removal of Motion Artifacts) (72); regressing out mean signals of gray matter, white matter, and cerebrospinal fluid tissues; and bandpass temporal filtering at 0.008 to 0.08 Hz (Figure S1).

Each brain was parcellated using the 400-region Schaefer cortical atlas (73) and the automated subcortical segmentation in FreeSurfer (74,75), producing a mean fMRI signal time series for each region. Then, we computed a Pearson’s correlation for every pair of these time series to generate a 419 × 419 FC matrix for each scan. For ΔFC-based predictions, matrices were instead derived by subtracting each patient’s baseline FC matrix from their 3-month FC matrix. This means that each entry in a patient’s ΔFC matrix contains their longitudinal ΔFC over the first 3 months for some coupling of brain regions. Patients without 3-month FC data were only included in models that used baseline FC as a predictor. Further details on acquisition parameters, quality control, and preprocessing are provided in the Supplement.

### Predictive Modeling

To evaluate the robustness of our findings, we predicted patient outcomes using 3 different algorithms: 1) connectome-based predictive modeling (CPM), 2) kernel ridge regression (KRR), and 3) multilayer meta-matching. The first two are commonly used in the field. The last has recently been shown to improve the performance of predictive models in small samples (76). All algorithms performed 4-fold cross-validation, where data are first divided into 4 equally sized folds. Three folds are used for training a model while the remaining fold is held aside for testing this model via prediction. This procedure is repeated 4 times, with each fold used for testing once. In the following section, we present details of each prediction algorithm.

#### Connectome-Based Predictive Modeling

CPM is a simple approach that prioritizes direct interpretability of brain regions and networks implicated in accurate predictions (77). We implemented CPM via 16 models, which differed in terms of the 1) outcome measure (symptoms or functioning), 2) outcome time point (6- or 12-month change), 3) predictor (baseline FC or ΔFC), and 4) feature selection (positive or negative). In each model, CPM first performs feature selection in the training set, using Pearson’s correlation to identify FC estimates that are either positively (*r* > 0, *p* < .05 uncorrected) or negatively (*r* < 0, *p* < .05 uncorrected) associated with outcomes. For each patient, FC values are then summed across these positive or negative features to create a summary score. A linear model is fitted between these FC summary scores and outcomes within the training set and then used to predict the outcomes of patients in the testing set.

#### Kernel Ridge Regression

KRR is a classical machine learning method that lacks a feature selection step, instead performing multivariable prediction with regularization, where a hyperparameter is optimized to control the trade-off between training error and testing error (78). We used the resting-state FC-based KRR implementation from Li *et al.* (79), which has been shown to predict many behavioral measures with accuracy comparable to that of more computationally demanding deep neural networks (80). KRR was implemented via 8 models, which differed in terms of the 1) outcome measure (symptoms or functioning), 2) outcome time point (6- or 12-month change), and 3) predictor (baseline FC or ΔFC). In KRR, each outcome in the testing set is predicted as a weighted mean of the outcomes observed in the training set. These weights include measures of similarity in FC between patients and an *l*_2_-regularization hyperparameter, the value of which is optimized via an inner loop of 4-fold cross-validation within the training set (further details are provided in the Supplement).

#### Multilayer Meta-Matching

Recent work suggests that prediction models that perform well within a small sample often fail to generalize across datasets (81, 82, 83, 84, 85). To boost our prediction accuracies while avoiding overoptimism, we also used multilayer meta-matching, a transfer learning framework that transposes prediction models trained on large healthy datasets to a smaller dataset of interest. This approach exploits neural degeneracy, whereby a relatively small set of resting-state FC patterns underlie many phenotypes spanning cognition, demographics, and mental health (86, 87, 88). We used the pretrained model described in Chen *et al.* (76), which significantly outperformed classical KRR in samples as small as 10 individuals and significantly predicted cognitive measures within and across multiple psychiatric cohorts (89).

Briefly, this involved using a deep neural network and linear ridge regression to predict 67 different phenotypes in the UK Biobank dataset (*n* = 36,834) (90) based on resting-state FC. These techniques were then used to predict another 162 phenotypes in other datasets [Adolescent Brain Cognitive Development, *n* = 5985 (91); Genomics Superstruct Project, *n* = 862 (92); Healthy Brain Network, *n* = 930 (93); Enhanced Nathan Kline Institute Rockland Sample, *n* = 896 (94)]. We used this pretrained model to predict all of these phenotypes for each STAGES patient, thereby generating 458 proxy variables that replaced FC as the inputs for a final KRR step to predict the clinical outcomes. Because no models have yet been pretrained using longitudinal changes in FC, we implemented meta-matching via 4 models, which differed in terms of the 1) outcome measure (symptoms or functioning) and 2) outcome time point (6- or 12-month change).

### Evaluating Prediction Performance and Significance

To further reduce sampling bias, we ran each of the above models across 100 splits, where each split used a different random allocation of patients to the 4 folds. Then, we quantified each model’s prediction performance as the mean correlation between predicted and observed outcomes across all 100 splits (*r_mean_*). We assessed prediction significance via permutation testing, which generates empirical null distributions of *r_mean_*. For each model, we used a fixed seed to randomly shuffle outcomes among patients 1000 times, and then prediction algorithms were rerun for each of these permutations. One hundred splits per permutation were used for CPM null models, but to reduce computational burden, we used 50 splits for KRR and 20 splits for meta-matching. We calculated *p* values as the proportion of null *r_mean_* values that exceeded the true *r_mean_*.

To correct for familywise error (FWE) arising from the inclusion of multiple predictive models, the inference-based Westfall-Young (or max statistic) method was chosen (95,96). To apply this method, we first grouped all null *r_mean_* values of each algorithm according to their permutation indices. For example, because we ran 16 CPM models, each of the 1000 permutations resulted in 16 null *r_mean_* values. Then, we selected the highest *r_mean_* at each permutation, producing a single null distribution for each of the 3 algorithms. These 3 FWE-corrected null distributions were then used to calculate all *p*_FWE_ values for the 16 CPM, 8 KRR, and 4 meta-matching models. By taking the maximum null value across all comparisons at each permutation, this method ensures that models only achieve significance by surpassing the strongest prediction performances across all null models that share the same prediction algorithm. We chose this approach to reduce false positives in a more tailored manner compared with alternative methods such as Bonferroni correction, which might have inflated the rate of false negatives (96,97). Statistical significance was assessed at *p*_FWE_ < .05.

## Results

### Clinical Outcomes

Across all patients included in our analysis, 76% had increased functioning (SOFAS) after the 6 months of STAGES treatment (mean proportional change = 20%, *n* = 49) (Figure S2), and 78% had increased functioning after 12 months (mean proportional change = 22%, *n* = 49). Similarly, 84% of patients had decreased symptoms (BPRS) after 6 months (mean proportional change = −25%, *n* = 5), and 87% had decreased symptoms after 12 months (mean proportional change = −23%, *n* = 45). Patients’ SOFAS scores at baseline (mean [SD] score = 52.5 [12.4]) (Table S1), 6 months (61.6 [16.1]), and 12 months (62.0 [10.6]) were comparable to those of a previously described naturalistic cohort of patients with FEP receiving antipsychotics and psychosocial interventions (*n* = 668 at baseline, ages 12–25 years) (98). Although BPRS total scores at baseline (57.8 [9.4]) [marked illness as per (99)], 6 months (42.6 [12.9]), and 12 months (43.0 [10.3]) were ∼20% higher than those of the same cohort, 6- and 12-month changes were comparable. Patients who discontinued prior to 3 months did not receive a second MRI scan, meaning that predictive models based on ΔFC used subsamples of 35 to 37 patients (Figure 1). Clinical outcomes did not differ significantly between patients with and those without 3-month FC data (2-tailed *t* test, *p* > .05 for all 6- and 12-month SOFAS and BPRS data).

### Predictive Modeling

#### Connectome-Based Predictive Modeling

**Figure 2:**
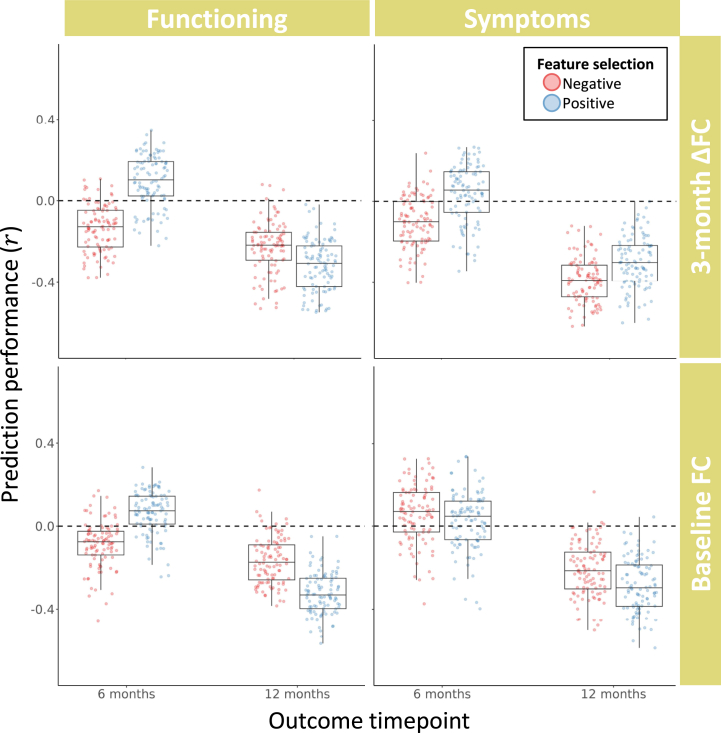
Performance of connectome-based predictive modeling for predicting changes in patients’ symptoms and functioning at 6 and 12 months using either baseline functional coupling (FC) or 3-month change in FC. Each data point shows the strength of Pearson’s correlation between predicted and observed clinical outcomes for a single split of 4-fold cross-validation, with each of the 16 models comprising 100 random splits.

The 8 CPM models that used baseline FC to predict outcomes showed limited predictive value (*r_mean_* range of −0.32 to 0.06) (Table 1 and Figure 2). This was also true for the 8 ΔFC models (*r_mean_* range of −0.39 to 0.10). Permutation testing via empirical null distributions revealed that none of the CPM models passed the threshold for statistical significance before or after FWE correction (all *p* ≥ .12) (Table 1 and Figure S3).

**Table 1:** Sample Size, Performance, and Significance for All 16 Connectome-Based Predictive Modeling Models

		Baseline FC				3-Month ΔFC			
		*n*	*r_mean_*	*p*	*p*_FWE_	*n*	*r_mean_*	*p*	*p*_FWE_
Functioning									
6 months	Neg	49	−0.09	.55	>.99	37	−0.14	.57	>.99
	Pos		0.06	.17	.91		0.10	.12	.84
12 months	Neg	49	−0.17	.69	>.99	36	−0.23	.73	>.99
	Pos		−0.32	.95	>.99		−0.32	.89	>.99
Symptoms									
6 months	Neg	45	0.06	.22	.92	36	−0.10	.45	>.99
	Pos		0.03	.24	.96		0.04	.20	.94
12 months	Neg	45	−0.21	.77	>.99	35	−0.39	.96	>.99
	Pos		−0.29	.88	>.99		−0.30	.87	>.99

FC, functional coupling; FWE, familywise error; Neg, negative feature model; Pos, positive feature model.

#### Kernel Ridge Regression

**Figure 3:**
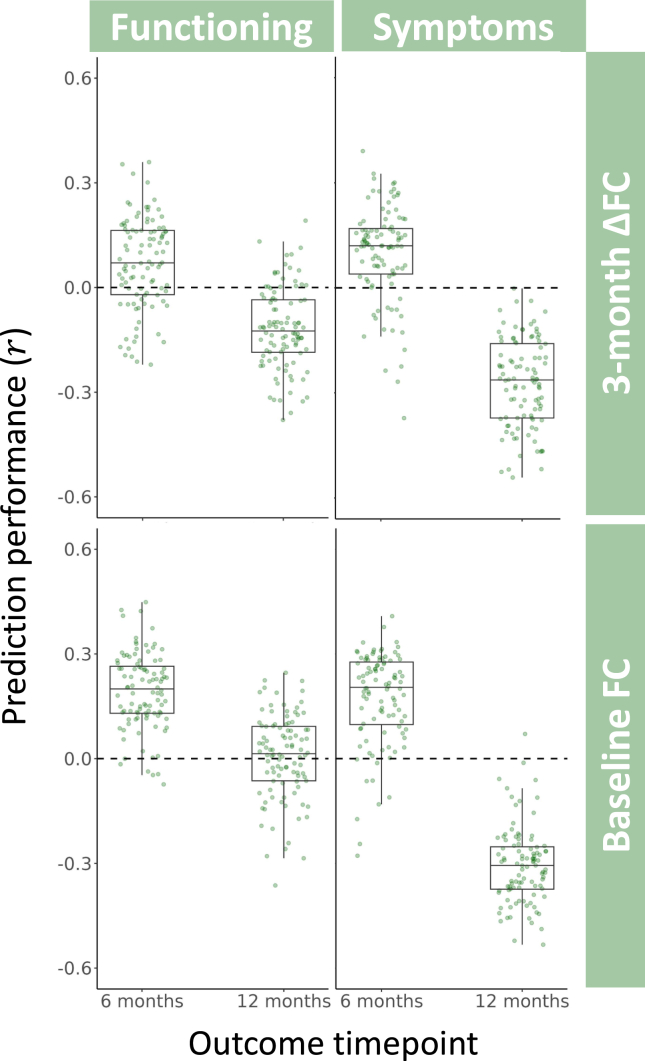
Performance of kernel ridge regression for predicting changes in patients’ symptoms and functioning at 6 and 12 months using either baseline functional coupling (FC) or 3-month change in FC. Each data point shows the strength of Pearson’s correlation between predicted and observed clinical outcomes for a single split of 4-fold cross-validation, with each of the 8 models comprising 100 random splits.

The 4 KRR models that used baseline FC to predict outcomes also showed limited predictive value (*r_mean_* range of −0.31 to 0.19) (Table 2 and Figure 3), as did the 4 ΔFC models (*r_mean_* range of −0.27 to 0.10). Permutation testing revealed that no KRR model passed the threshold for statistical significance before or after FWE correction (all *p* ≥ .14) (Table 2 and Figure S4).

**Table 2:** Sample Size, Performance, and Significance for All 8 Kernel Ridge Regression Models

	Baseline FC				3-Month ΔFC			
	*n*	*r_mean_*	*p*	*p*_FWE_	n	*r_mean_*	*p*	*p*_FWE_
Functioning								
6 months	49	0.19	.14	.74	37	0.07	.37	.96
12 months	49	0.01	.50	>.99	36	−0.12	.67	>.99
Symptoms								
6 months	45	0.17	.19	.78	36	0.10	.33	.94
12 months	45	−0.31	.97	>.99	35	−0.27	.92	>.99

FC, functional coupling; FWE, familywise error.

#### Multilayer Meta-Matching

**Figure 4:**
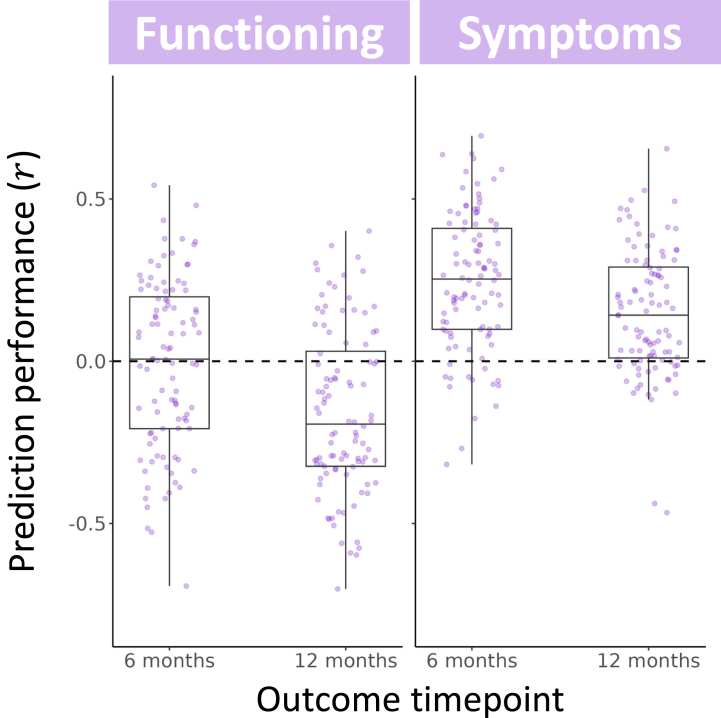
Performance of multilayer meta-matching for predicting changes in patients’ symptoms and functioning at 6 and 12 months using baseline functional coupling. Each data point shows the strength of Pearson’s correlation between predicted and observed clinical outcomes for a single split of 4-fold cross-validation, with each of the 4 models comprising 100 random splits.

The 4 multilayer meta-matching models that used baseline FC to predict outcomes also demonstrated limited performance (*r_mean_* range of −0.16 to 0.24) (Table 3 and Figure 4), and none achieved significance before or after FWE correction (all *p* ≥ .09) (Table 3 and Figure S5).

**Table 3:** Sample Size, Performance, and Significance for All 4 Multilayer Meta-Matching Models

	Baseline FC			
	*n*	*r_mean_*	*p*	*p*_FWE_
Functioning				
6 months	49	−0.01	.53	.94
12 months		−0.16	.83	>.99
Symptoms				
6 months	45	0.24	.09	.29
12 months		0.15	.21	.58

FC, functional coupling; FWE, familywise error.

#### Exploratory Models

To account for temporal variability in clinical assessments, we explored quantifying each patient’s outcomes as slopes of symptoms or functioning over 12 months, using simple linear regressions fitted to their available BPRS/SOFAS performance (*r_mean_* < 0.3) (Figure S6 and Table S2). We also explored predicting changes in patients’ positive symptoms, derived by summing only 4 items of the BPRS (unusual thought content, conceptual disorganization, hallucinatory behavior, and grandiosity), and found poor performance (*r_mean_* < 0.3) (Figure S7 and Table S3).

Poor performance was generally observed when predicting the 6-month outcomes of placebo (*n* = 14) and medication (*n* = 22 to 35) groups separately (*r_mean_* < 0.3) (Figures S8–S13 and Tables S4–S6). In this analysis, the medication group included 6 patients who discontinued placebo treatment and had substantial exposure to antipsychotics by 6 months (>1750 mg chlorpromazine equivalent). For some models, baseline FC moderately predicted placebo patient functioning (*r_mean_* = 0.34 and 0.49 for KRR and meta-matching, respectively) and symptoms (*r_mean_* = 0.37 for meta-matching). However, performance varied substantially due to the small sample size, with many splits reaching *r* < 0, and no model achieved significance (*p* ≥ .05).

The robustness of our main results is affirmed by model performances remaining low (*r_mean_* < 0.3) when we excluded gray matter signal regression from fMRI preprocessing (Figures S14–S16 and Tables S7–S9), using an alternative 328-region parcellation (*p* ≥ .12) (Figures S17–S20; Tables S10 and S11) or alternative feature selection thresholds of *p* < .05/.001 in CPM (*p* ≥ .06) (Figures S21–S24; Tables S12 and S13).

## Discussion

Many fMRI studies have reported disrupted FC across the psychosis spectrum by comparing patients with healthy control participants (21,23, 24, 25, 26, 27, 28, 29, 30, 31), but it remains unclear whether FC holds potential as a prognostic biomarker that can predict treatment outcomes in FEP. We leveraged the long-term longitudinal measurement of symptoms and functioning from a clinical trial, together with gold-standard prediction algorithms, to evaluate whether resting-state FC could predict outcomes of patients with FEP at the end of the 6-month trial and after 12 months.

All the CPM, KRR, and meta-matching algorithms showed poor prediction performance (all *r_mean_* < 0.3, except for 3 exploratory models within the placebo group, although with highly variable performance) for all outcome measures, and none of the models achieved statistical significance. Our results contrast with those of previous FEP prediction studies, where FC either predicted treatment response compared with nonresponse (defined via a priori symptom thresholds) with balanced accuracies exceeding 75% (45,46,48,49) or significantly predicted longitudinal changes in symptoms (44,47,48,50). Several differences between the previous and current work may explain this difference. First, our sample of patients with FEP included the full spectrum of psychotic disorders, whereas most previous studies only included patients diagnosed with schizophrenia. Second, patients in our sample received psychosocial interventions alongside either placebo or antipsychotic tablets, whereas previous studies generally involved more rigid treatment protocols (e.g., all patients received similar antipsychotic dosing with no psychosocial interventions). Third, we considered whole-brain FC, whereas most previous studies defined a priori predictors localized to specific regions or networks. Predictions were consequently driven by different FC patterns across studies, including cerebello-thalamo-cortical (47), cortico-cortical (44,48), and hippocampal-cortical (45) circuits, as well as FC seeded in the bilateral anterior cingulate cortex (50) and striatum (46).

Together with our null findings, this body of work suggests that FC may only predict clinical outcomes within highly selected samples and contexts and may not generalize to the heterogeneous samples and treatment settings encountered in real-world clinics (100,101). It is also possible that FC may only capture a patient’s capacity for clinical changes during the early phase of treatment, given that the only other study to predict outcomes beyond 16 weeks also reported the lowest performance (*r* = 0.21) among previous studies that used continuous outcomes (47). We chose to predict continuous outcomes to preserve meaningful variance between patients but acknowledge that dichotomization is vital to clinical decision making and therefore necessary for personalizing care. This is particularly relevant for positive psychotic symptoms, where antipsychotics are effective (102) and the Andreasen criteria provide a consensus definition of remission (103). Because outcomes are not typically distributed bimodally, future research validating prognostic biomarkers should maximize statistical power and analytic flexibility by dichotomizing after predicting continuous outcomes, using thresholds relevant to the clinical decisions being considered.

Our previous study of this cohort used CPM with baseline measures of structural connectivity to significantly predict 12-month changes in functioning (*r_mean_* = 0.44), significantly outperforming predictive models based on patients’ baseline clinical, cognitive, and demographic data (22). This result suggests that diffusion MRI–derived estimates of structural connectivity hold greater potential as a prognostic biomarker in FEP than resting-state FC, which has lower test-retest reliability (104) and correlates with a range of in-scanner internal states (105, 106, 107) and self-reported experiences (108,109). However, prognostic trait-like FC patterns may be revealed by estimating FC during movie watching (110) or across multiple task and rest conditions (111, 112, 113, 114) [see Cao *et al.* for FEP outcome prediction based on such cross-paradigm FC (44)].

### Limitations

Although our sample size of 35 to 49 per main prediction model was similar to previous studies, it may be underpowered for capturing the full range of possible FC and clinical outcomes or robust predictive modeling (81, 82, 83, 84, 85). This is particularly pertinent for the exploratory predictions of placebo and medication group outcomes, where models relied on 14 to 35 individuals. We included meta-matching to compensate for the small sample size, but this approach only improves prediction performance compared with single-sample methods when the phenotype of interest is strongly correlated with at least 1 phenotype from the large source datasets used in training (76,115). To our knowledge, no previous studies have used meta-matching for longitudinal prediction, and so patients’ clinical outcomes might not have been closely related to any of the 229 phenotypes in the 5 cross-sectional source datasets. While changes in symptoms and functioning were comparable to those of a large naturalistic cohort of patients with FEP (98), the extensive exclusion criteria required to include a placebo arm in the trial might have biased FC measures, potentially diminishing some associations with outcomes. Therefore, the reliability and generalizability of our finding that resting-state FC did not predict clinical outcomes of patients with FEP should be assessed by performing cross-validation on a large, representative sample (83,100,101). However, this remains challenging due to the paucity of open-access FEP neuroimaging datasets with longitudinal outcomes.

The BPRS, which we used to measure symptom severity, has demonstrated lower interrater reliability, internal consistency, and clinical predictive power than other scales that are more burdensome to administer (116,117). This might have weakened our statistical power for detecting associations between FC and symptom changes and explain the poor prediction performance compared with studies that used other scales (47, 48, 49). Effect sizes may also be maximized via factor analytic techniques (118,119).

Heterogeneity in clinical outcomes among patients with FEP can also be explained by non-neurobiological factors, including baseline symptoms and functioning (4,5,10,101,120, 121, 122, 123, 124, 125, 126, 127), age (4,121,128,129), sex (4,10,124,126), schizotypal traits (10,121), inflammatory markers (130,131), education and employment status (123,124,129,132), substance use (123,126,133,134), recent life events (135), previous depressive episodes (124), social environment (124,136), and duration of untreated psychosis (4, 5, 6,120, 121, 122,127,137, 138, 139, 140, 141, 142). If these factors do not covary with neuroimaging measures, their effects will impose an upper limit on the performance and clinical utility of brain-based prognostic biomarkers. It is important that future work evaluates how the combination of different biological, demographic, and clinical measures affects prediction of outcomes (143, 144, 145). Given the expense and complexity of fMRI, promising FC-based prognostic biomarkers should demonstrate predictive capacity beyond that afforded by simpler measures (101,122,146,147).

### Conclusions

Our analysis, using multiple cross-validated prediction algorithms, indicates that neither brainwide resting-state FC at baseline nor FC change over 3 months significantly predicts changes in symptoms or functioning of patients with FEP 6 to 12 months after commencing treatment. When taken together with past work, this finding suggests that FC may only hold prognostic utility within narrow clinical and experimental contexts.

## Supplementary Material

Supplemental Text, Figures S1–S24, and Tables S1–S13

Key Resources Table
