# Supplementary material for: Functional Coupling and Longitudinal Outcome Prediction in First-Episode Psychosis
Source: Biol Psychiatry Glob Open Sci. 2025 Aug 11;5(6):100589. doi: 10.1016/j.bpsgos.2025.100589 (PMC12495094; doi:10.1016/j.bpsgos.2025.100589)
Supplement: Supplemental Text, Figures S1–S24, and Tables S1–S13 [file mmc1.pdf]

SUPPLEMENTARY INFORMATION

Functional Coupling and Longitudinal Outcome Prediction in First-Episode Psychosis

Pope *et al.*

Supplement

Supplementary Information – STAGES clinical trial details

Supplementary Information – MRI acquisition, quality control, and preprocessing details

Supplementary Information – Further information on kernel ridge regression

Supplementary Table 1 – Sample characteristics for included patients

Supplementary Figure 1 – Quality control metrics throughout fMRI preprocessing

Supplementary Figure 2 – Clinical outcomes for included patients

Supplementary Figure 3 – Null distribution for the 16 CPM models

Supplementary Figure 4 – Null distribution for the 8 KRR models

Supplementary Figure 5 – Null distribution for the 4 meta-matching models

Supplementary Figure 6 – Performance of all algorithms when predicting slopes of symptoms/functioning across timepoints

Supplementary Table 2 – Sample size and performance of models predicting slopes of symptoms/functioning across timepoints

Supplementary Figure 7 – Performance of all algorithms when predicting changes in BPRS positive symptoms

Supplementary Table 3 – Sample size and performance of models predicting changes in BPRS positive symptoms

Supplementary Figure 8 – Performance of CPM when predicting placebo/medication group outcomes separately

Supplementary Figure 9 – Null distribution for CPM when predicting placebo/medication group outcomes separately

Supplementary Table 4 – Sample size, performance, and significance of CPM models predicting placebo/medication group outcomes separately

Supplementary Figure 10 – Performance of KRR predicting placebo/medication group outcomes separately

Supplementary Figure 11 – Null distribution for KRR when predicting placebo/medication group outcomes separately

Supplementary Table 5 – Sample size, performance, and significance of KRR models predicting placebo/medication group outcomes separately

Supplementary Figure 12 – Performance of meta-matching when predicting placebo/medication group outcomes separately

Supplementary Figure 13 – Null distribution for meta-matching when predicting placebo/medication group outcomes separately

Supplementary Table 6 – Sample size, performance, and significance of meta-matching models predicting placebo/medication group outcomes separately

Supplementary Figure 14 – Performance of CPM when excluding grey matter signal regression from fMRI preprocessing

Supplementary Table 7 – Sample size and performance of CPM models excluding grey matter signal regression from fMRI preprocessing

Supplementary Figure 15 – Performance of KRR when excluding grey matter signal regression from fMRI preprocessing

Supplementary Table 8 – Sample size and performance of KRR models excluding grey matter signal regression from fMRI preprocessing

Supplementary Figure 16 – Performance of meta-matching when excluding grey matter signal regression from fMRI preprocessing

Supplementary Table 9 – Sample size and performance of meta-matching models excluding grey matter signal regression from fMRI preprocessing

Supplementary Figure 17 – Performance of CPM with alternative parcellation

Supplementary Figure 18 – Null distribution for CPM with alternative parcellation

Supplementary Table 10 – Sample size, performance, and significance of CPM models with alternative parcellation

Supplementary Figure 19 – Performance of KRR with alternative parcellation

Supplementary Figure 20 – Null distribution for KRR with alternative parcellation

Supplementary Table 11 – Sample size, performance, and significance of KRR models with alternative parcellation

Supplementary Figure 21 – Performance of CPM with alternative parcellation and feature selection at $p < .05$

Supplementary Figure 22 – Null distribution for CPM with alternative parcellation and feature selection at $p < .05$

Supplementary Table 12 – Sample size, performance, and significance of CPM models with alternative parcellation and feature selection at $p < .05$

Supplementary Figure 23 – Performance of CPM with alternative parcellation and feature selection at $p < .001$

Supplementary Figure 24 – Null distribution for CPM with an alternative parcellation and feature selection at $p < .001$

Supplementary Table 13 – Sample size, performance, and significance of CPM models with alternative parcellation and feature selection at $p < .001$

STAGES clinical trial details

To maximize safety and comply with standards set by the Melbourne Health Human Research and Ethics Committee, potential participants were subject to the following exclusion criteria: (i) inability to provide informed consent; (ii) risk to self or others (score of 5 or greater on the BPRS-4 Suicidality and Hostility subscales); (iii) duration of untreated psychosis greater than 6 months; (iv) pregnancy; (v) non-negligible prior use of antipsychotics outside the trial (7 days or 1750 mg chlorpromazine equivalent) or present use of mood stabilizers; and (vi) unstable accommodation or support. Study discontinuation criteria also included these items, along with (vii) substantial exacerbation of positive symptoms (2-point increase on the BPRS-4 subscale for Conceptual Disorganization, Hallucinations, Unusual Thought Content, or Suspiciousness, maintained for 1 week and not due to substance use); (viii) substantial and sustained drop in overall functioning (20-point decrease on the SOFAS from baseline, maintained for 1 month); (ix) request by the participant for antipsychotic medication; and (x) minimal recovery after 3 months (>4 on the BPRS-4 Hallucinations, Suspiciousness, and Unusual Thought Content subscales, or >3 on Conceptual Disorganization).

Participants were allocated to antipsychotic and placebo arms of the trial in a 1:1 ratio, using a randomization design that stratified for sex and the duration of untreated psychosis (0-30, 31-90, >90 days). Participants, clinicians, and research staff remained blinded to this allocation throughout the trial. Participants in the antipsychotic arm were administered either 1 mg risperidone or 3 mg paliperidone, depending on recruitment period. Multiple compounds were used due to changing availabilities of matched placebo tablets, with these dosages converted using olanzapine equivalent units. All participants received cognitive behavioral case management, comprising manualized cognitive behavioral therapy and psychoeducation, aimed at preventing relapse and enhancing coping strategies for positive and negative symptoms (1). Additional support via group programs and advocacy was provided by case managers. Clinicians assessed participants' BPRS and SOFAS scores at each session. After the 6-month treatment period, all participants were offered open-label treatment at the same clinic. Participants who discontinued the trial were offered to remain in cognitive behavioral case management and receive antipsychotic medication as deemed appropriate by the treating team. The full study protocol can be found in O'Donoghue et al. (2), with trial outcomes reported by Francey et al. (3).

MRI acquisition, quality control, and preprocessing details

Participants were instructed to maintain wakefulness and remain still while lying in the scanner with their eyes open. For each anatomical scan, interleaved acquisition was used to capture 176 slices under the following parameters: repetition time = 2300ms; echo time = 2.98ms; flip angle of 9°; FOV of 256mm; voxel size of 1.1 x 1.1. x 1.2 mm. For each functional scan, interleaved acquisition was used to capture 37 slices per volume, for 234 volumes, under the following parameters: repetition time = 2000ms; echo time = 32ms; flip angle = 90°; field of view = 210mm; slice thickness of 3.5 mm, and 3.3 x 3.3 x 3.55 mm voxels.

A total of 97 scans from baseline and 3 months were first evaluated by the quality control software MRI-QC, which automatically estimates 56 no-reference image quality metrics for T1-weighted scans and 31 metrics for functional scans. By providing both individual- and group-level visualizations of these metrics, MRI-QC allows the identification

of any outlier scans with issues such as motion artefacts, signal spillover, coil artefacts, poor contrast, and signal dropout (4). We excluded from further analysis two baseline and one 3-month scan with such problems. The 3-month counterpart to one of these baseline scans was also excluded, as longitudinal change in functional coupling (FC) cannot be estimated from a single scan. Remaining scans were preprocessed using the standard automated pipeline in fMRIPrep v1.4.1 (5) as follows.

First, anatomical images were corrected for signal intensity non-uniformity via the N4BiasFieldCorrection algorithm, to improve tissue contrast across the brain (6). All 176 slices per scan were then spatially aligned to each other in a common orientation using the Advanced Normalization Tools (ANTs) package (7). Images were also skull-stripped with ANTs, prior to tissue segmentation using FMRIB's Automated Segmentation Tool (8). FreeSurfer (9) was used to reconstruct pial, grey matter, and white matter surfaces from these tissue maps. Anatomical volumes were then transformed to the standard MNI152NLin6Asym space (10) using ANTs. Separately, all 37 slices per functional volume were spatially aligned to each other, then all 234 volumes per scan were spatially aligned to each other. This corrects for in-scanner head motion, with brain realignment parameters estimated in 6 time series (translation and rotation in 3 dimensions each) by the MCFLIRT function in the FMRIB software library (11). To adjust for the asynchronous acquisition of slices, the AFNI package performed slice-time correction (12), which temporally shifts the blood oxygen level-dependent (BOLD) signal time series of each slice to adjust for the asynchronous acquisition of slices. This step aligns with recommendations by Sladky et al. (2011), who found robust increases in statistical power for repetition times of 2 seconds or longer (13). To adjust for inhomogeneities in the applied magnetic field, which are known to stretch, compress, and shift image voxels (14), ANTs performed susceptibility distortion estimation and correction. FreeSurfer then aligned all functional volumes to the anatomical volumes.

In-scanner head motion has been shown to distort blood oxygen level dependent (BOLD) signal time series, with spatially heterogenous effects leading to spurious increases and decreases in estimates of FC (15–17). We used the method from Jenkinson et al. (2002) to calculate each fMRI scan's time series of framewise displacement (FD) (11), which quantified the total amount of head movement between each of the 234 volumes acquired. We then classified and excluded one high-motion scan according to the stringent criteria in Parkes et al. (2018) (18), which advises to exclude a scan if it meets any of the following: (i) $\text{mean FD} > 0.25\text{ mm}$; (ii) $20\%\text{ of FD} > 0.2\text{ mm}$; and (iii) any single $\text{FD} > 5\text{ mm}$. The 92 remaining scans (55 at baseline, 37 at 3 months) displayed low head motion ($\text{mean FD} = 0.049\text{ mm}, \text{SD} = 0.020\text{ mm}$) compared to large resting-state fMRI datasets of the general population (18).

Since fMRI has an inherently low signal-to-noise ratio, FC-based analyses typically include denoising beyond the minimal steps in fMRIPrep (18). We chose a denoising pipeline that has been shown to minimize three quality control benchmarks (18): (i) FD-FC correlations, which quantify how head motion impacts each FC estimate across all scans; (ii) FD-FC distance dependence, which quantifies how FD-FC correlations change with the coupling distance that separates each pair of regions; and (iii) temporal degrees of freedom lost by denoising, which confers reduced statistical power for analyses.

In each scan, we first applied linear detrending to the BOLD signal time series of every voxel. Scans were then denoised with independent component analysis for the automatic removal of motion artefacts (ICA-AROMA). ICA-AROMA identifies motion-related spatial components of the fMRI data based on a priori thresholds for spatial features (overlap with cerebrospinal fluid, overlap with pial surface) and a linear discriminant analysis for temporal features (correlation with head motion, high-frequency content) (19). In line with recommendations (19,20), smoothed fMRI data (kernel of 6 mm full-width at half-maximum) were input to ICA-AROMA. To mitigate the impacts of head motion on FC estimates, the time series of these motion-related components were then non-aggressively regressed out from every voxel in the non-smoothed detrended scan. Here, non-aggressive regression refers to performing regression while preserving any temporal variance shared between regressors (i.e., motion-related components) and signal (i.e., grey matter) time series, and was used in the original ICA-AROMA paper (19). To reduce the impact of any non-neural fluctuations (e.g., cardiac, respiratory) on FC estimation, we also regressed out mean signals of the white matter and cerebrospinal fluid tissues (21). These signals were calculated using conservative tissue masks, derived by repeatedly eroding the tissue probability maps from fMRIPrep (5 erosion cycles for white matter, 2 for cerebrospinal fluid).

One common yet controversial denoising step in resting-state fMRI preprocessing is global signal regression, which removes the mean whole-brain signal from every voxel (22). Since it centers each scan's FC distribution around zero, global signal regression introduces potentially spurious anti-correlations. Although the global signal partially reflects non-neural physiology and head motion (18,21,23), it also has behaviorally-relevant neuronal contributions (24–26). Importantly, global signal regression tends to improve behavioral prediction (27), justifying its inclusion in our study. Since the global signal is strongly correlated with the mean grey matter signal (22), we used grey matter signal regression as a substitute that may avoid reintroducing noise by regressing out white matter and cerebrospinal fluid signals twice (28). Grey matter tissue masks were derived by thresholding the corresponding fMRIPrep probability maps at 70%. As per recommendations (15,29), we also regressed out the temporal derivative (calculated as backward differences), the square, and the square of the temporal derivative for each of the white matter, cerebrospinal fluid, and grey matter time series. To avoid reintroducing noise, we calculated all tissue signals after ICA-AROMA denoising and performed regressions simultaneously (30). We also include exploratory prediction models where no grey matter signal regression was performed. To further isolate neural signal, we applied a band-pass filter of $f = 0.008\text{-}0.08\ Hz$ to the BOLD signal time series.

We parcellated each scan's grey matter using the 400-region cortical atlas from Schaefer et al. (31) and the automated subcortical segmentation in FreeSurfer (32,33). To adjust for individual differences in cortical anatomy, the Schaefer atlas was mapped onto each participant's grey matter using manually-corrected tissue surfaces from FreeSurfer. Regional BOLD signal time series were calculated as a mean across all voxels in each of the 419 regions, weighting each voxel's contribution by its fMRIPrep-estimated probability of being grey matter. An alternative 328-region parcellation was also tested for the CPM and KRR models (Figures S, comprised by the Schaefer300 (31) and Scale II Melbourne subcortical atlas (34). We excluded the 4 globi pallidi regions from this alternative parcellation due to weak signal

intensity, identified using the largest gap (or 'elbow') in regions' average signal intensities as a threshold (35). Each scan's FC matrix was constructed by taking Pearson's correlation between the BOLD time series of each pair of regions.

FD-FC correlations were computed for all FC estimates in the 328-region parcellation after each denoising step, shown as box plots in Figure S1a. Despite the minimal head motion, moderate FD-FC correlations were present after fMRIPrep ($\text{mean}\ r = 0.11, \text{SD} = 0.12$), and were exacerbated by linear detrending ($\text{mean}\ r = 0.16, \text{SD} = 0.12$). Contrary to its intended purpose, ICA-AROMA had little impact on FD-FC correlations ($\text{mean}\ r = 0.17, \text{SD} = 0.11$), which were instead reduced by tissue-based regressions (8Phys-4GMR; $\text{mean}\ r = 0.014, \text{SD} = 0.13$) and band-pass frequency filtering ($\text{mean}\ r = 0.0061, \text{SD} = 0.12$). Moderate FD-FC distance dependence was present after fMRIPrep ($\rho = 0.19$; Figure S1b). Most subsequent denoising steps did not mitigate FD-FC distance dependence ($\rho = 0.21 - 0.22$), except band-pass filtering which slightly reduced the association ($\rho = 0.17$).

Further information on kernel ridge regression

Each kernel ridge regression model randomly divided patients into four folds, three of which formed the training set of N individuals. For each individual s in the testing fold, their clinical outcome y_s was predicted as a weighted mean of the outcomes observed in the training set (contained in the $\boldsymbol{y}^{train}$ vector):

$$y_s = \boldsymbol{K}_s(\boldsymbol{K} + \lambda \boldsymbol{I})^{-1}\boldsymbol{y}^{\text{train}} \quad (1)$$

Here, $\boldsymbol{I}$ denotes the $N \times N$ identity matrix and $\boldsymbol{K}$ denotes the $N \times N$ matrix containing strengths of Pearson's correlation between every pair of vectorized upper-triangle FC matrices in the training set. That is, the $(i, j)^{th}$ element of $\boldsymbol{K}$ is given by:

$$(\boldsymbol{K})_{ij} = \text{corr}\left(FC_i^{\text{train}}, FC_j^{\text{train}}\right) \quad (2)$$

$\boldsymbol{K}_s$ similarly denotes the $1 \times N$ vector containing strengths of Pearson's correlation between the vectorized upper-triangle FC matrix of individual s and those in the training set:

$$(\boldsymbol{K}_s)_i = \text{corr}\left(FC_s, FC_i^{\text{train}}\right) \quad (3)$$

A range of values for the l_2-regularization hyperparameter λ were tested via an inner loop of 4-fold cross-validation within the training set. The value of λ which best minimised a cost function across individuals was then input in Equation 1 to predict outcomes in the testing set. More information is available at:

https://github.com/ThomasYeoLab/CBIG/tree/master/utilities/matlab/predictive_models/KernelRidgeRegression

Table S1: Baseline sample characteristics for STAGES patients included in the present study.

	**First-episode psychosis patients ($n = 55$)**
Baseline age, years (SD)	19.24 (2.89)
Females, N (%)	28 (50.9%)
Left handedness, N (%)	3 (5.5%)
Education, years (SD)	12.22 (2.17)
Diagnosis, N	
Major depression with psychosis	11
Schizophreniform disorder	8
Psychotic disorder not otherwise specified	14
Substance-induced psychotic disorder	6
Delusional disorder	5
Schizophrenia	10
Missing diagnosis	1
Baseline BPRS total, mean (SD)	56.76 (9.71)
Baseline SOFAS, mean (SD)	52.49 (12.42)
Baseline SANS, mean (SD)	34.07 (17.41)
Baseline HAM-D, mean (SD)	19.89 (5.27)
Baseline HAM-A, mean (SD)	20.73 (6.63)
Baseline QLS, mean (SD)	70.45 (20.62)

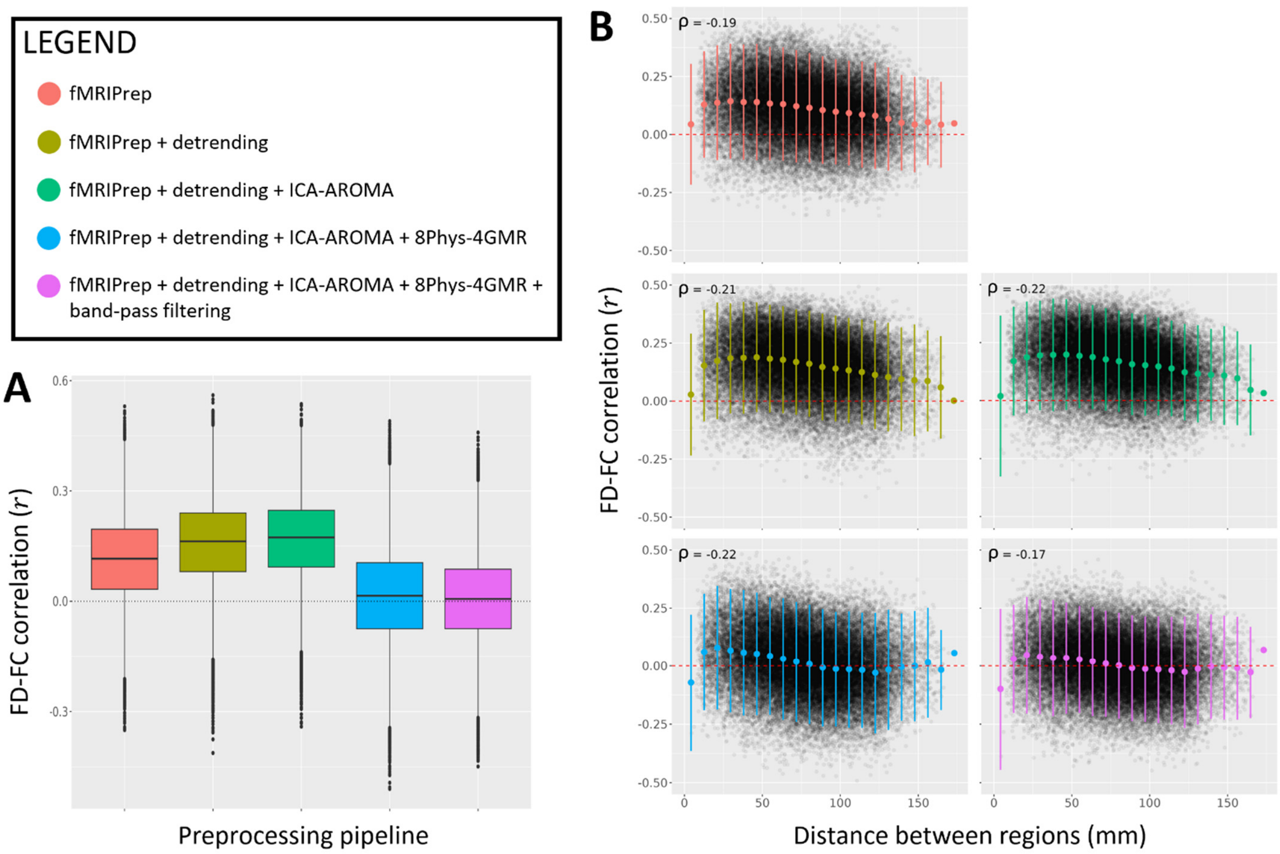

Figure S1: Quality control metrics quantifying the impact of in-scanner head motion on functional coupling (FC) estimates, assessed throughout preprocessing with the 328-region parcellation. **(a)** Correlations between framewise displacement (FD) and FC, computed after each denoising step outlined in the legend. Each data point represents the FD-FC correlation for some pair of regions, across all scans. **(b)** FD-FC correlations plotted against the coupling distance that separates each pair of regions. Each plot was computed after a different denoising step, with mean and standard deviation bars colored according to the legend. The FD-FC distance dependence is indicated by Spearman's rho on each plot. '8Phys-4GMR' refers to the tissue-based regressions.

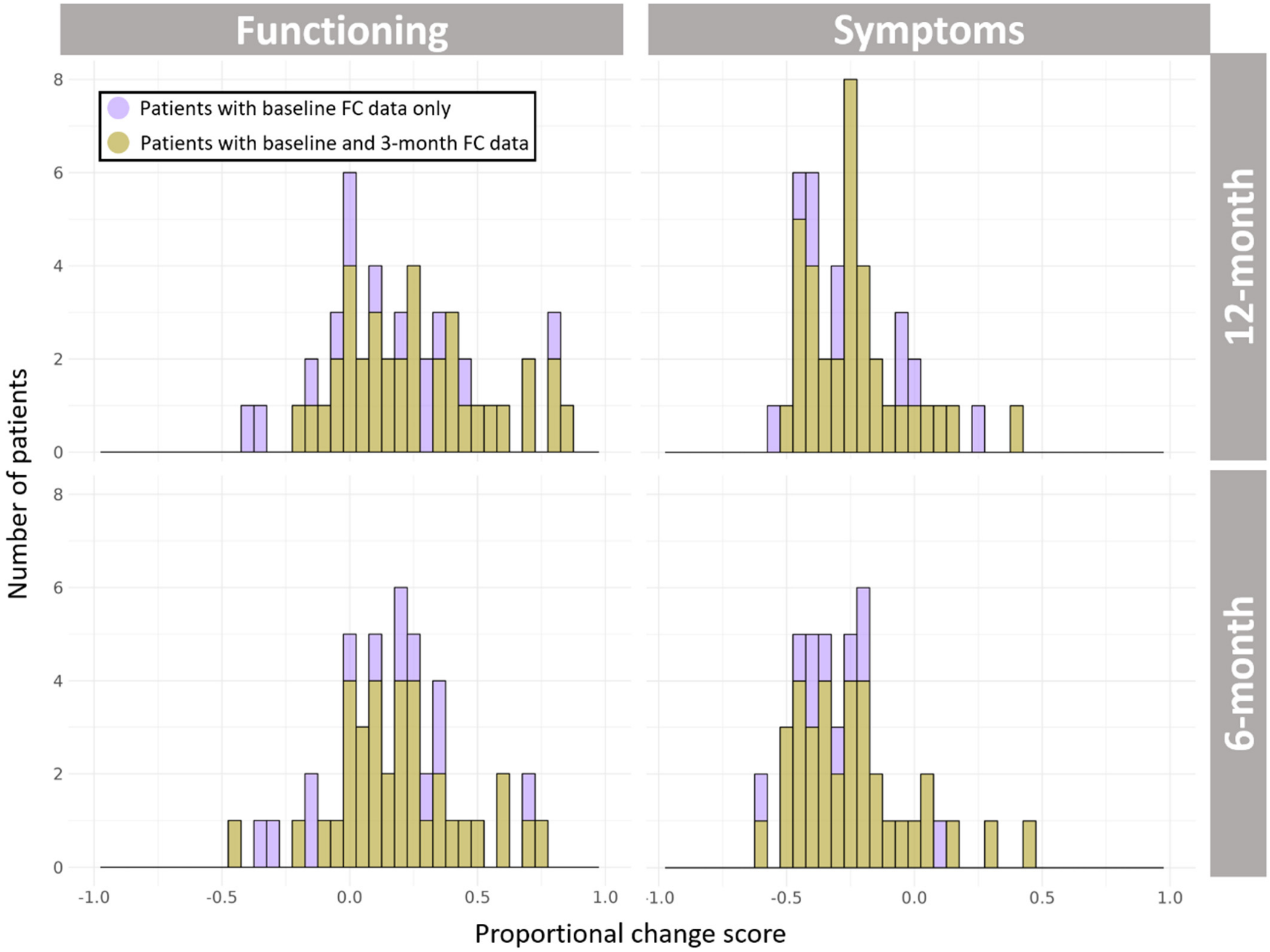

Figure S2: Clinical outcomes for patients included in the present study, defined as proportional changes in total SOFAS and BPRS scores after 6 and 12 months. FC, functional coupling.

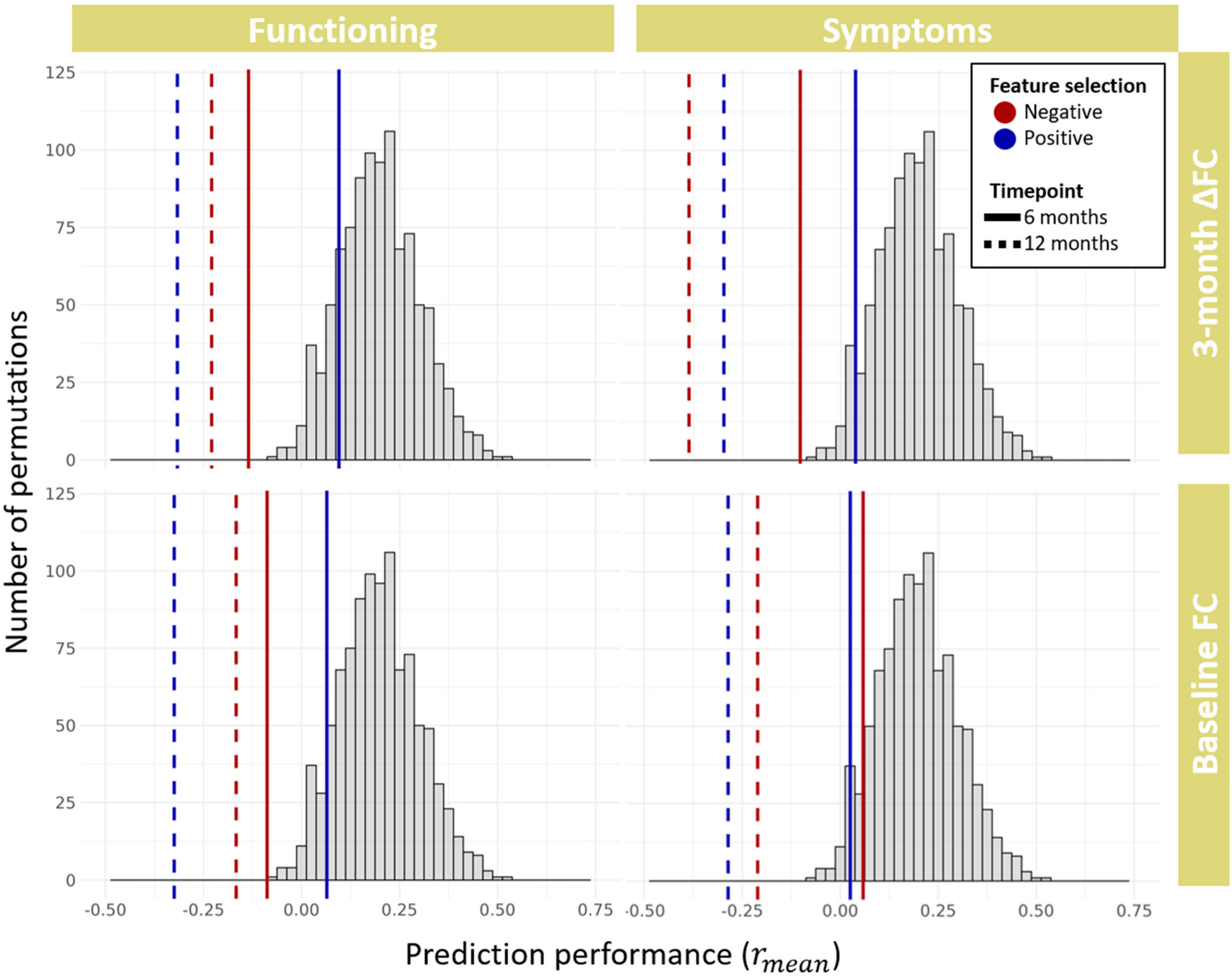

Figure S3: Prediction performance (r_{mean}) for connectome-based predictive modelling (CPM), shown in blue and red, superimposed against a family-wise error (FWE)-corrected empirical null distribution derived by randomly permuting clinical outcomes amongst patients. For each of 1000 permutations, 100 splits of CPM were run to calculate a single null r_{mean}. FC, functional coupling.

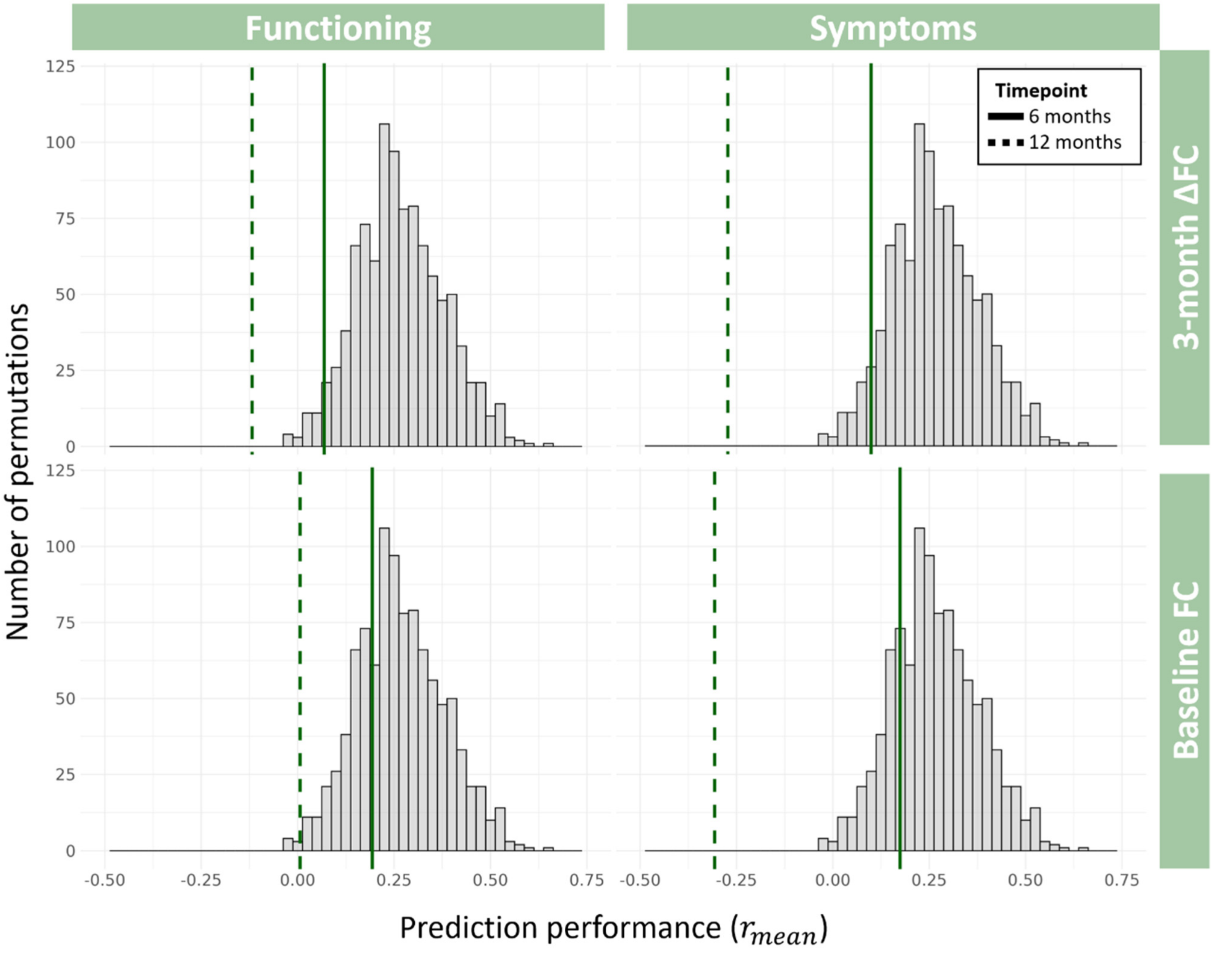

Figure S4: Prediction performance (r_{mean}) for kernel ridge regression (KRR), shown in green, superimposed against a family-wise error (FWE)-corrected empirical null distribution derived by randomly permuting clinical outcomes amongst patients. For each of 1000 permutations, 50 splits of KRR were run to calculate a single null r_{mean}. FC, functional coupling.

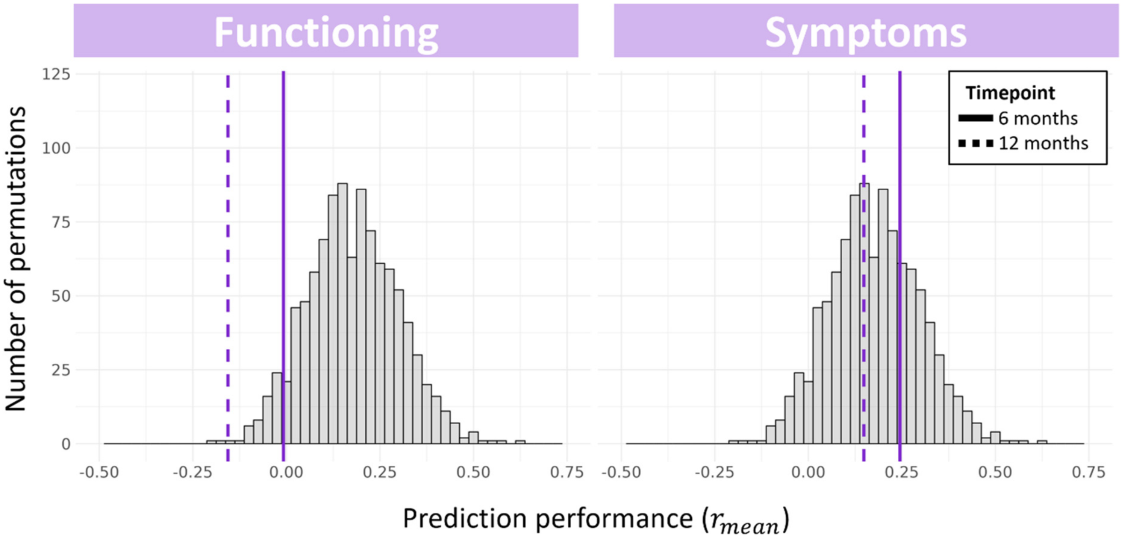

Figure S5: Prediction performance (r_{mean}) for multilayer meta-matching, shown in purple, superimposed against a family-wise error (FWE)-corrected empirical null distribution derived by randomly permuting clinical outcomes amongst patients. For each of 1000 permutations, 20 splits of multilayer meta-matching were run to calculate a single null r_{mean}.

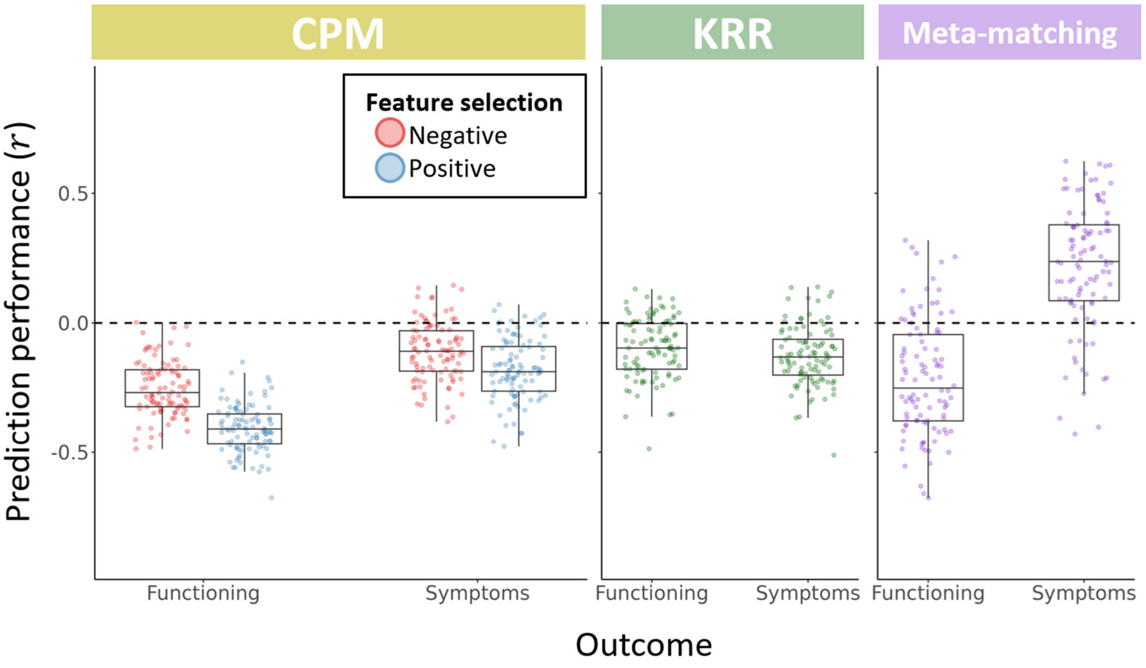

Figure S6: Performance of all algorithms using baseline functional coupling (FC) to predict patients' slopes of functioning and symptoms over time, derived from simple linear regressions fitted to their baseline and 12-month SOFAS/BPRS scores, as well as 1-, 3-, and 6-month scores where available. Each data point shows the strength of Pearson's correlation between predicted and observed clinical outcomes for a single split of 4-fold cross-validation, with each of the 8 models comprising 100 random splits. CPM, connectome-based predictive modelling; KRR, kernel ridge regression.

Table S2: Sample size and performance for exploratory models using baseline functional coupling (FC) to predict patients' slopes of symptoms and functioning over time. CPM, connectome-based predictive modelling; pos, positive feature model; neg, negative feature model; KRR, kernel ridge regression.

		**Symptoms** **($n = 45$)**	**Functioning** **($n = 49$)**
**Baseline FC**		r_{mean}	r_{mean}
CPM	*neg*	-0.25	-0.11
	pos	-0.41	-0.18
KRR		-0.09	-0.13
Meta-matching		-0.22	0.22

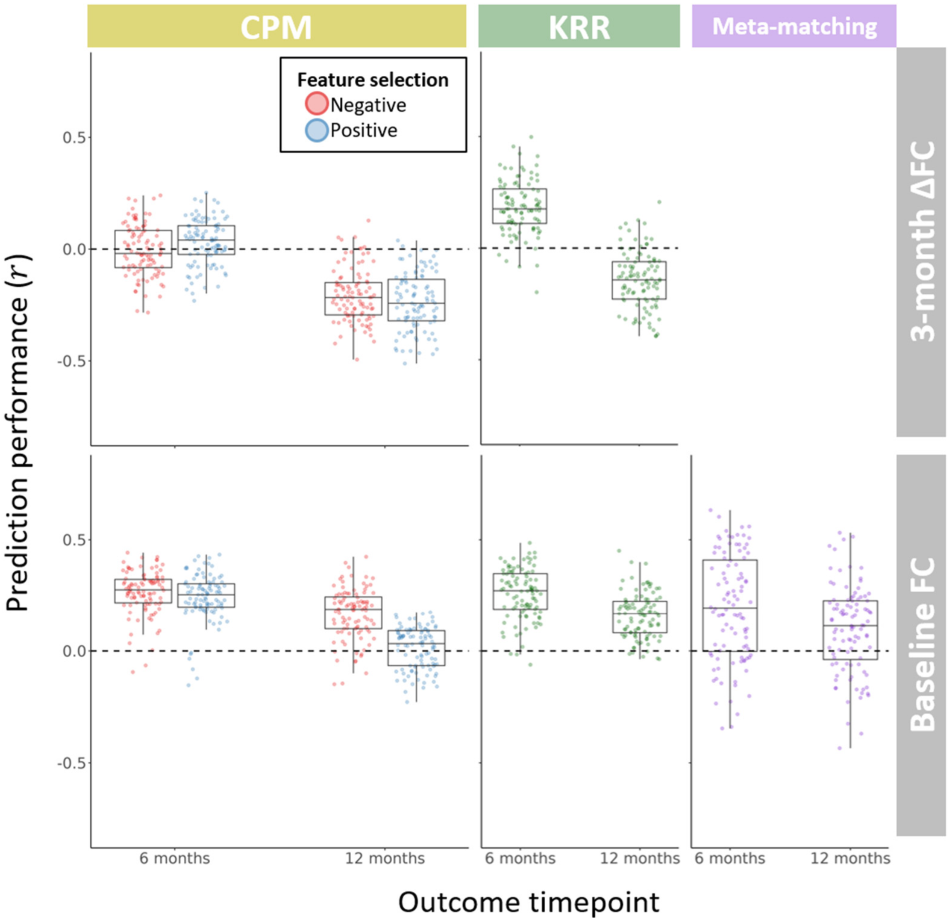

Figure S7: Performance of all algorithms for predicting patients' change scores on the Brief Psychiatric Rating Scale (BPRS) positive symptom subscale. Each data point shows the strength of Pearson's correlation between predicted and observed clinical outcomes for a single split of 4-fold cross-validation, with each of the 16 models comprising 100 random splits. CPM, connectome-based predictive modelling; KRR, kernel ridge regression; FC, functional coupling.

Table S3: Sample size and performance for exploratory models predicting changes in Brief Psychiatric Rating Scale (BPRS) positive symptoms. CPM, connectome-based predictive modelling; pos, positive feature model; neg, negative feature model; KRR, kernel ridge regression; FC, functional coupling.

<table>
<tr><th colspan="2"></th><th colspan="2">Baseline FC</th><th colspan="2">3-month ΔFC</th></tr>
<tr><th colspan="2">6 months</th><th>n</th><th>r_{mean}</th><th>n</th><th>r_{mean}</th></tr>
<tr><td rowspan="2">CPM</td><td>neg</td><td rowspan="4">45</td><td>0.26</td><td rowspan="3">36</td><td>-0.01</td></tr>
<tr><td>pos</td><td>0.24</td><td>0.03</td></tr>
<tr><td colspan="2">KRR</td><td>0.26</td><td>0.18</td></tr>
<tr><td colspan="2">Meta-matching</td><td>0.19</td><td>--</td><td>--</td></tr>
<tr><th colspan="2">12 months</th><th>n</th><th>r_{mean}</th><th>n</th><th>r_{mean}</th></tr>
<tr><td rowspan="2">CPM</td><td>neg</td><td rowspan="4">45</td><td>0.17</td><td rowspan="3">35</td><td>-0.21</td></tr>
<tr><td>pos</td><td>0.01</td><td>-0.24</td></tr>
<tr><td colspan="2">KRR</td><td>0.16</td><td>-0.14</td></tr>
<tr><td colspan="2">Meta-matching</td><td>0.10</td><td>--</td><td>--</td></tr>
</table>

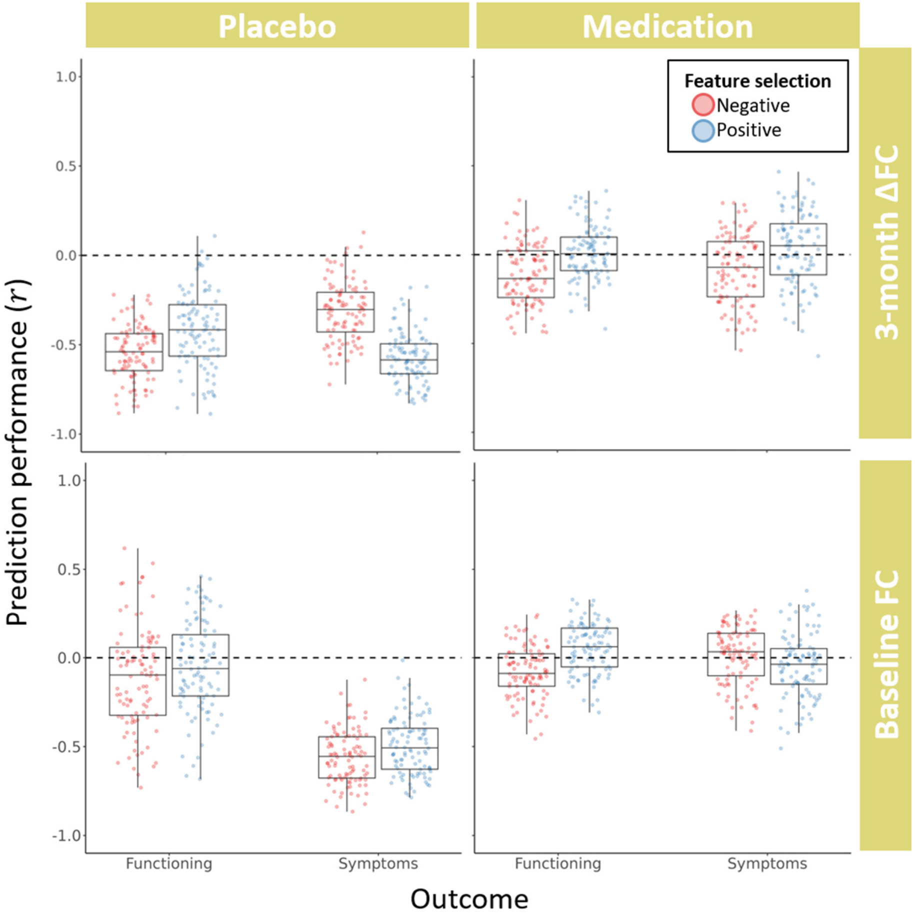

Figure S8: Performance of connectome-based predictive modelling (CPM) for predicting 6-month outcomes of placebo and medication groups separately. Each data point shows the strength of Pearson's correlation between predicted and observed clinical outcomes for a single split of 4-fold cross-validation, with each of the 16 models comprising 100 random splits. FC, functional coupling.

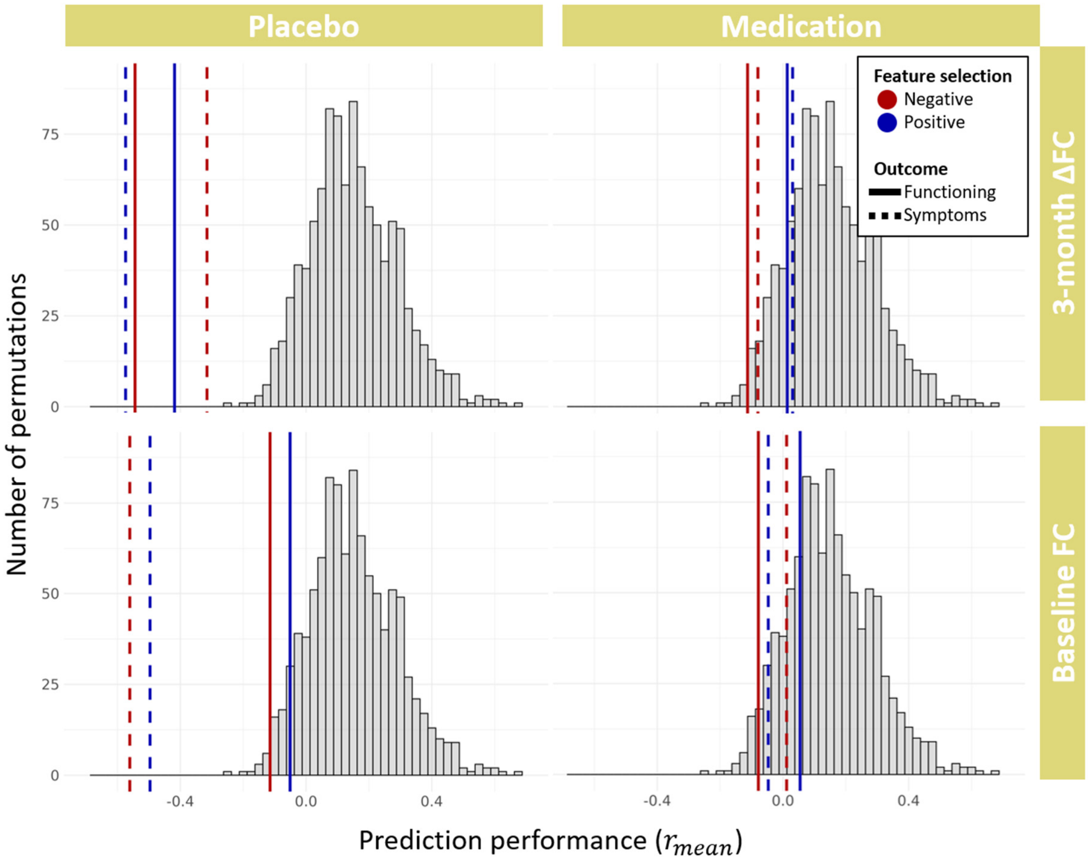

Figure S9: Prediction performance (r_{mean} shown in red and blue) when using connectome-based predictive modelling (CPM) to predict 6-month outcomes of placebo and medication groups separately. Model performances are superimposed against a family-wise error (FWE)-corrected empirical null distribution derived by randomly permuting clinical outcomes amongst patients. For each of 1000 permutations, 100 splits of CPM were run to calculate a single null r_{mean}. FWE correction was applied across the 16 models shown. FC, functional coupling.

Table S4: Sample size, performance, and significance for exploratory connectome-based predictive modelling (CPM) models predicting 6-month outcomes of placebo and medication groups separately. pos, positive feature model; neg, negative feature model; FC, functional coupling.

		Baseline FC				3-month ΔFC			
**Functioning**		n	r_{mean}	p	p_{FWE}	n	r_{mean}	p	p_{FWE}
Placebo	*neg*	14	-0.11	.21	.99	14	-0.54	.90	>.99
	pos		-0.05	.15	.94		-0.42	.67	>.99
Medication	*neg*	35	-0.08	.42	.97	23	-0.11	.36	.99
	pos		0.05	.19	.76		0.01	.24	.85
**Symptoms**		n	r_{mean}	p	p_{FWE}	n	r_{mean}	p	p_{FWE}
Placebo	*neg*	14	-0.56	.91	>.99	14	-0.32	.42	>.99
	pos		-0.50	.82	>.99		-0.57	.94	>.99
Medication	*neg*	31	0.01	.24	.85	22	-0.08	.29	.97
	pos		-0.05	.34	.94		0.03	.15	.81

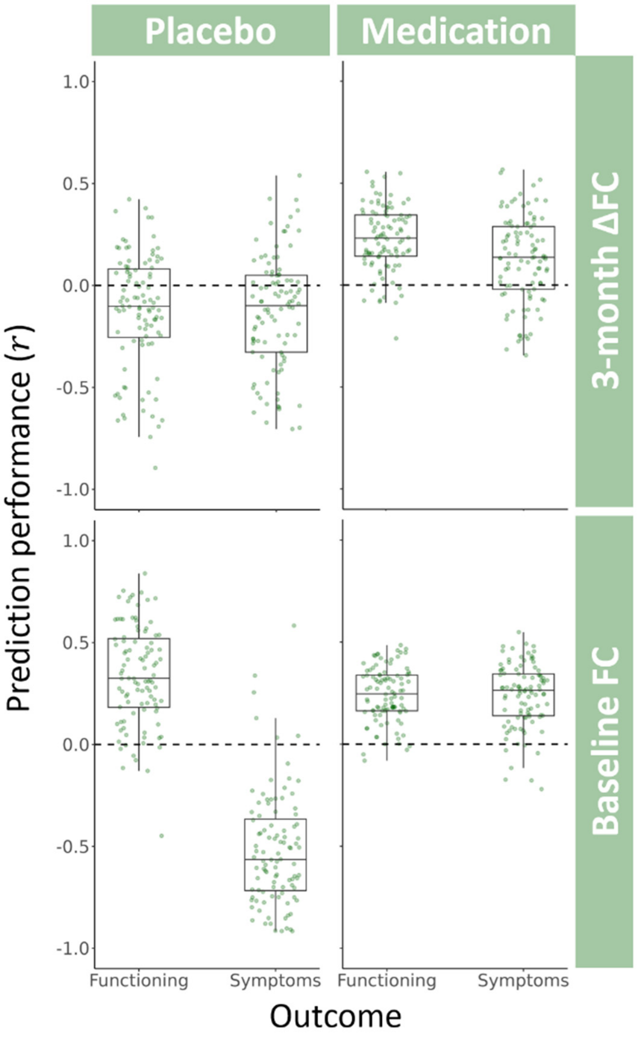

Figure S10: Performance of kernel ridge regression (KRR) for predicting 6-month outcomes of placebo and medication groups separately. Each data point shows the strength of Pearson's correlation between predicted and observed clinical outcomes for a single split of 4-fold cross-validation, with each of the 8 models comprising 100 random splits. FC, functional coupling.

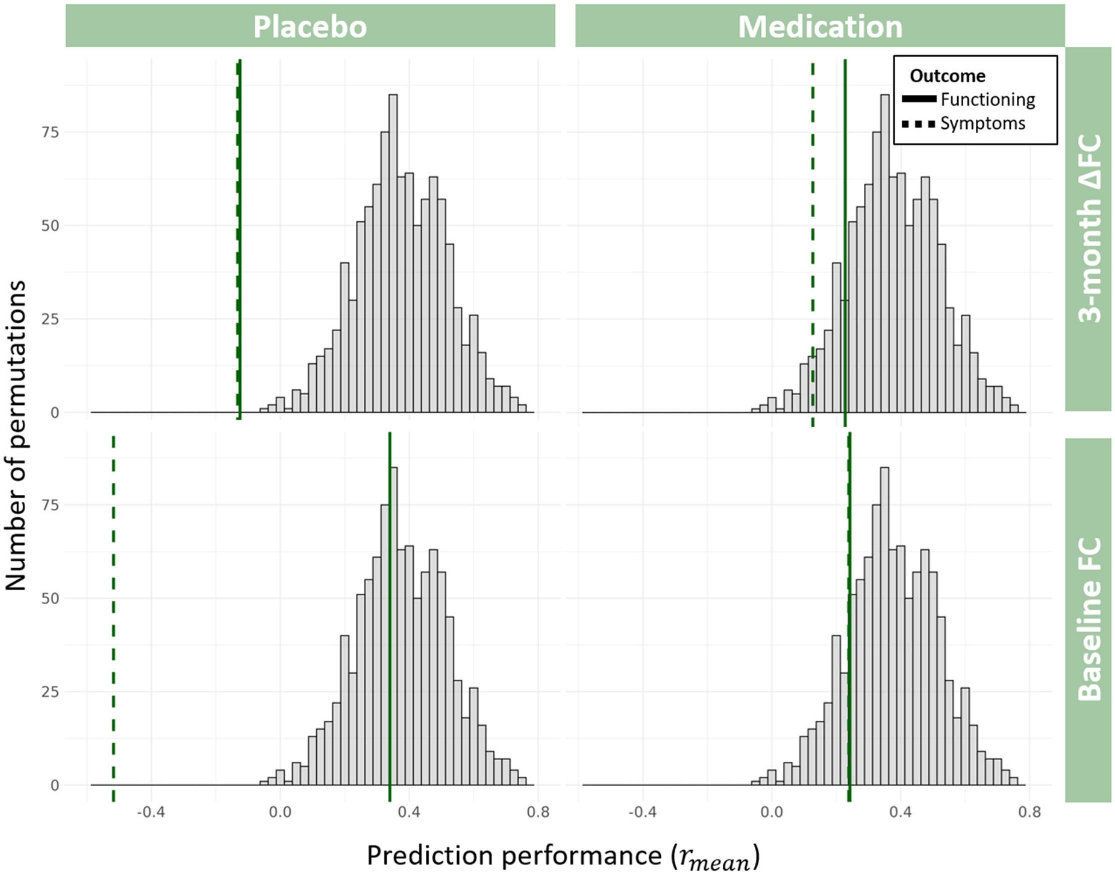

Figure S11: Prediction performance (r_{mean} shown in green) when using kernel ridge regression (KRR) to predict 6-month outcomes of placebo and medication groups separately. Model performances are superimposed against a family-wise error (FWE)-corrected empirical null distribution derived by randomly permuting clinical outcomes amongst patients. For each of 1000 permutations, 50 splits of KRR were run to calculate a single null r_{mean}. FWE correction was applied across the 8 models shown. FC, functional coupling.

Table S5: Sample size, performance, and significance for exploratory kernel ridge regression (KRR) models predicting 6-month outcomes of placebo and medication groups separately. FC, functional coupling.

	**Baseline FC**				**3-month ΔFC**			
**Functioning**	n	r_{mean}	p	p_{FWE}	n	r_{mean}	p	p_{FWE}
Placebo	14	0.34	.13	.59	14	-0.12	.66	>.99
Medication	35	0.24	.12	.84	23	0.23	.21	.86
**Symptoms**	n	r_{mean}	p	p_{FWE}	n	r_{mean}	p	p_{FWE}
Placebo	14	-0.52	.96	>.99	14	-0.13	.66	>.99
Medication	31	0.24	.15	.84	22	0.13	.33	.96

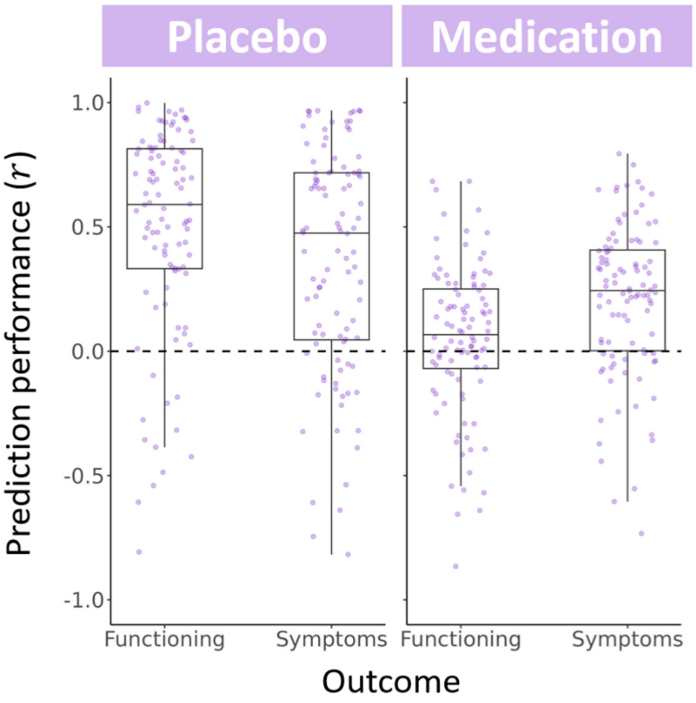

Figure S12: Performance of multilayer meta-matching for predicting 6-month outcomes of placebo and medication groups separately. Each data point shows the strength of Pearson's correlation between predicted and observed clinical outcomes for a single split of 4-fold cross-validation, with each of the 4 models comprising 100 random splits. FC, functional coupling.

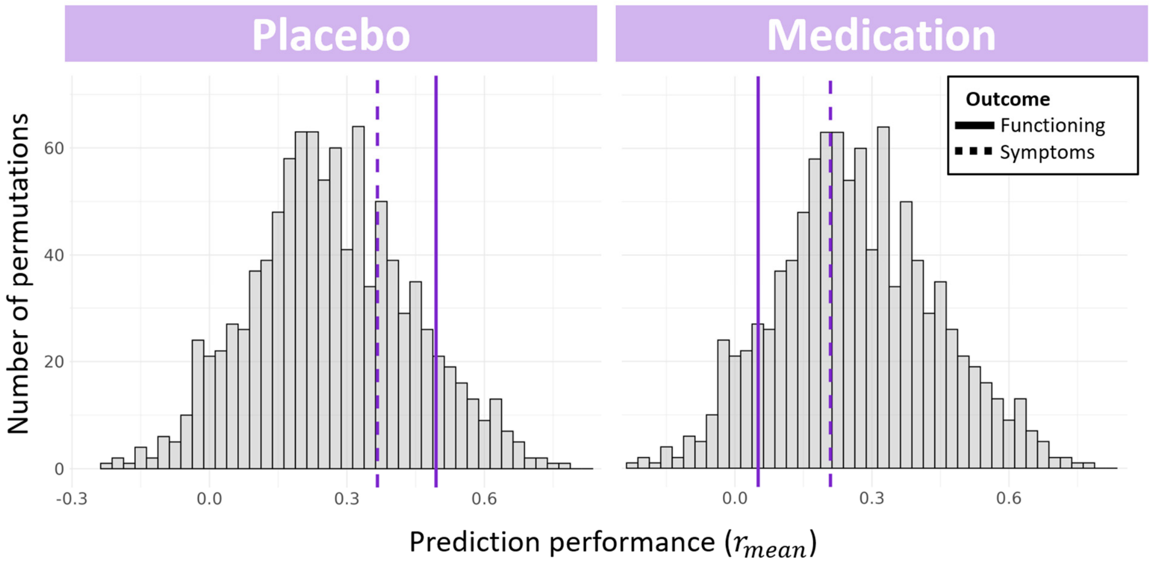

Figure S13: Prediction performance (r_{mean} shown in purple) when using multilayer meta-matching to predict 6-month outcomes of placebo and medication groups separately. Model performances are superimposed against a family-wise error (FWE)-corrected empirical null distribution derived by randomly permuting clinical outcomes amongst patients. For each of 1000 permutations, 20 splits of meta-matching were run to calculate a single null r_{mean}. FWE correction was applied across the 4 models shown. FC, functional coupling.

Table S6: Sample size, performance, and significance for exploratory multilayer meta-matching models predicting 6-month outcomes of placebo and medication groups separately. FC, functional coupling.

	Baseline FC			
**Functioning**	n	r_{mean}	p	p_{FWE}
Placebo	14	0.49	.05	.10
Medication	35	0.05	.40	.89
**Symptoms**	n	r_{mean}	p	p_{FWE}
Placebo	14	0.37	.13	.28
Medication	31	0.21	.17	.61

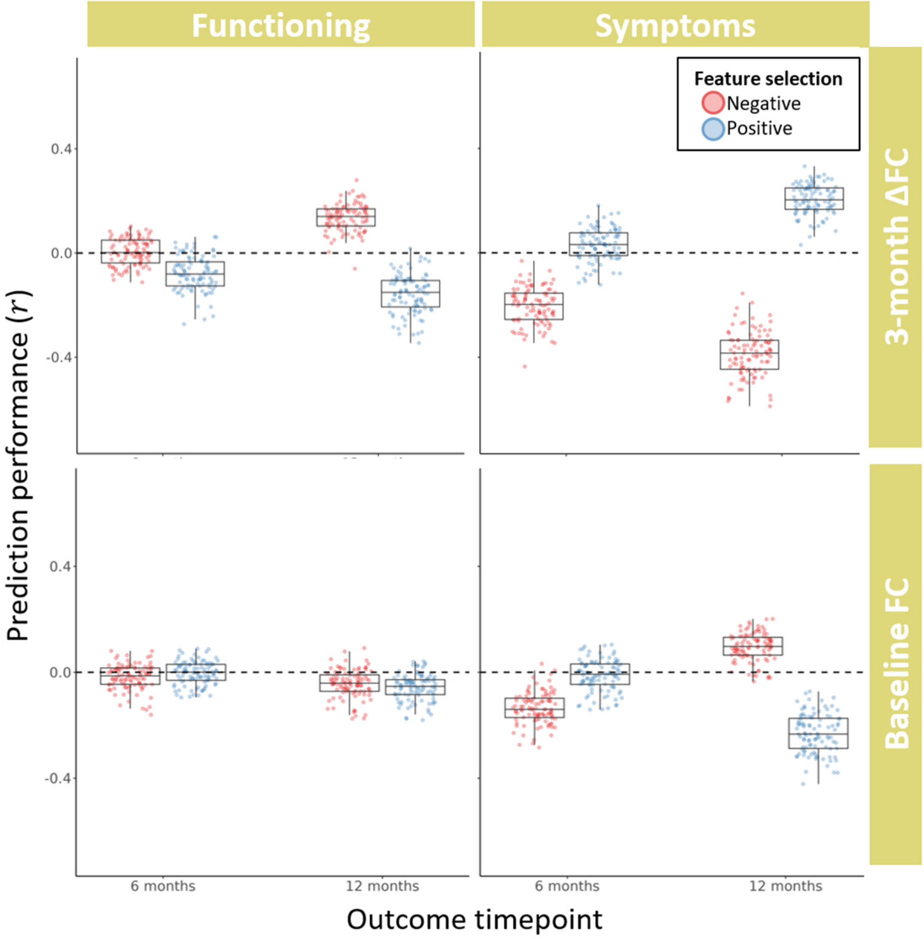

Figure S14: Performance of connectome-based predictive modelling (CPM) for predicting patients' clinical outcomes, using functional coupling (FC) estimates derived without performing grey matter signal regression. Each data point shows the strength of Pearson's correlation between predicted and observed clinical outcomes for a single split of 4-fold cross-validation, with each of the 16 models comprising 100 random splits.

Table S7: Sample size and performance for exploratory connectome-based predictive modelling (CPM) models excluding grey matter signal regression in functional MRI preprocessing. pos, positive feature model; neg, negative feature model; FC, functional coupling.

		**Baseline FC**		**3-month ΔFC**	
**Functioning**		n	r_{mean}	n	r_{mean}
6-month	*neg*	49	-0.02	37	0.00
	pos		0.00		-0.08
12-month	*neg*	49	-0.04	36	0.14
	pos		-0.06		-0.16
**Symptoms**		n	r_{mean}	n	r_{mean}
6-month	*neg*	45	-0.14	36	-0.20
	pos		-0.01		0.03
12-month	*neg*	45	0.10	35	-0.39
	pos		-0.23		0.20

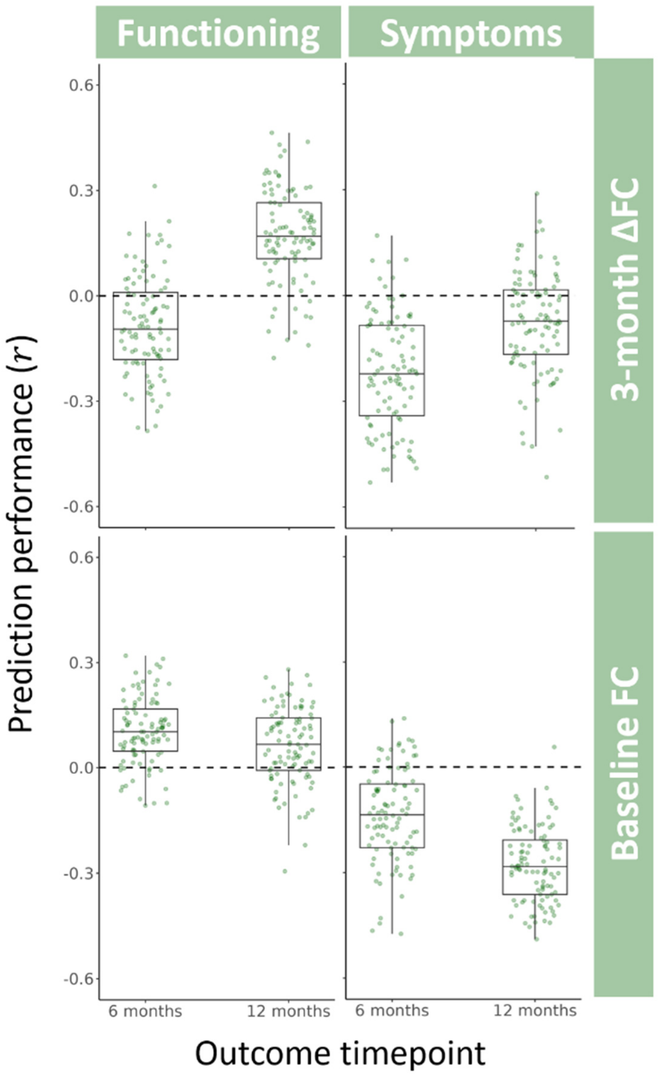

Figure S15: Performance of kernel ridge regression (KRR) for predicting patients' clinical outcomes, using functional coupling (FC) estimates derived without performing grey matter signal regression. Each data point shows the strength of Pearson's correlation between predicted and observed clinical outcomes for a single split of 4-fold cross-validation, with each of the 8 models comprising 100 random splits.

Table S8: Sample size and performance for exploratory kernel ridge regression (KRR) models excluding grey matter signal regression in functional MRI preprocessing. FC, functional coupling.

	**Baseline FC**		**3-month ΔFC**	
**Functioning**	n	r_{mean}	n	r_{mean}
6-month	49	0.10	37	-0.09
12-month	49	0.06	36	0.17
**Symptoms**	n	r_{mean}	n	r_{mean}
6-month	45	-0.14	36	-0.22
12-month	45	-0.28	35	-0.08

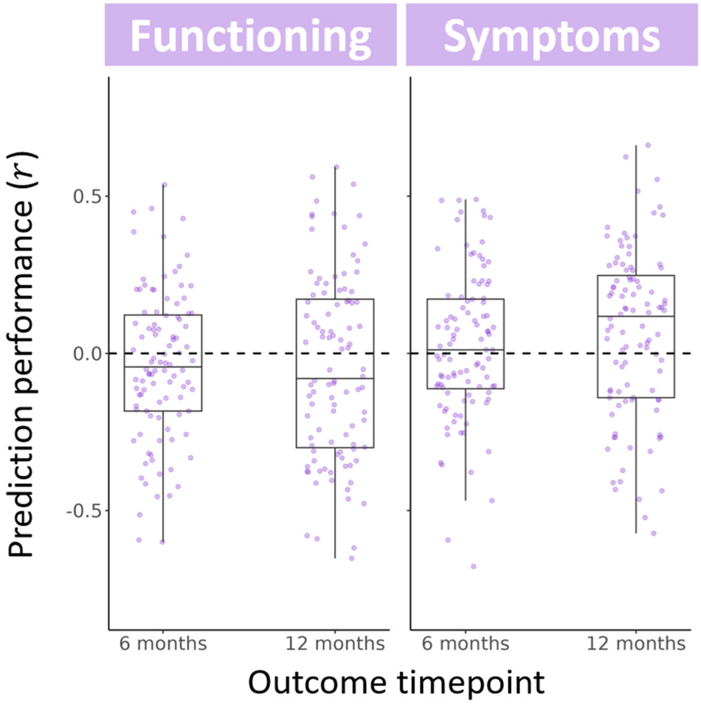

Figure S16: Performance of multilayer meta-matching for predicting patients' clinical outcomes, using functional coupling estimates derived without performing grey matter signal regression. Each data point shows the strength of Pearson's correlation between predicted and observed clinical outcomes for a single split of 4-fold cross-validation, with each of the 4 models comprising 100 random splits.

Table S9: Sample size and performance for exploratory multilayer meta-matching models excluding grey matter signal regression in functional MRI preprocessing. FC, functional coupling.

	Baseline FC	
**Functioning**	n	r_{mean}
6-month	49	-0.04
12-month		-0.05
**Symptoms**	n	r_{mean}
6-month	45	0.02
12-month		0.06

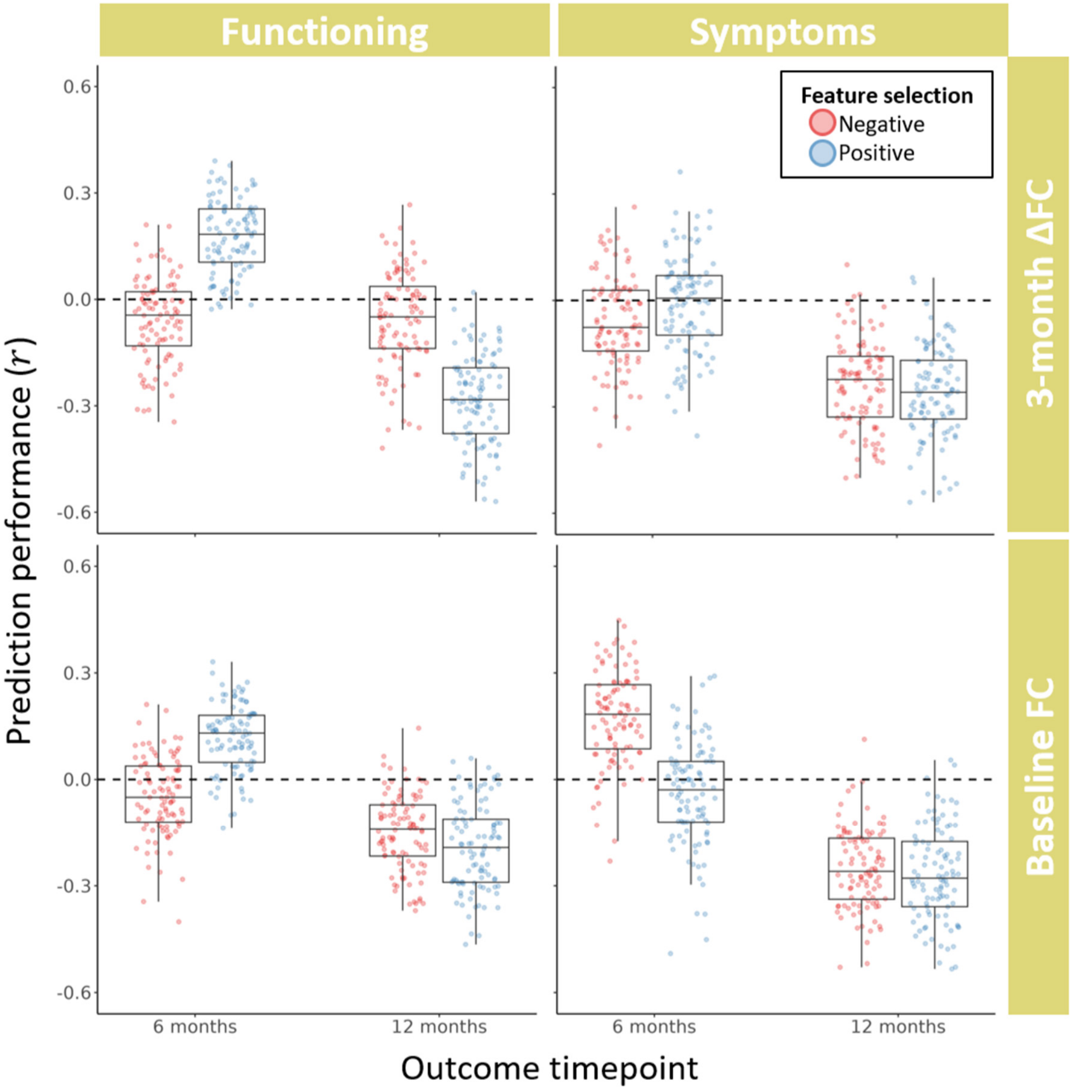

Figure S17: Performance of connectome-based predictive modelling (CPM) for predicting patients' clinical outcomes, using the 328-region parcellation. Each data point shows the strength of Pearson's correlation between predicted and observed clinical outcomes for a single split of 4-fold cross-validation, with each of the 16 models comprising 100 random splits. FC, functional coupling.

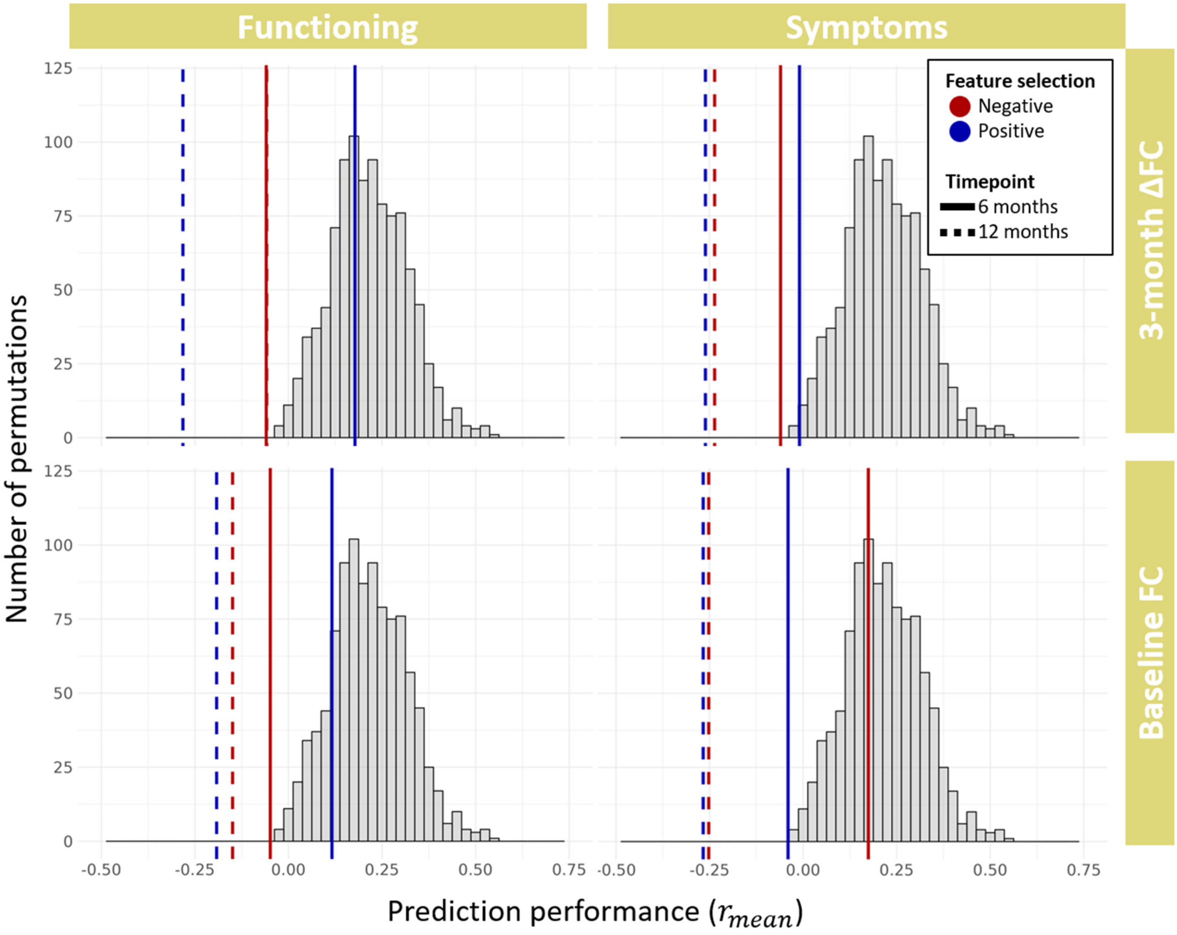

Figure S18: Prediction performance (r_{mean} shown in red and blue) for all 16 connectome-based predictive modelling (CPM) models, using the 328-region parcellation. Model performances are superimposed against a family-wise error (FWE)-corrected empirical null distribution derived by randomly permuting clinical outcomes amongst patients. For each of 1000 permutations, 100 splits of CPM were run to calculate a single null r_{mean}. FWE correction was applied across the 16 models shown. FC, functional coupling.

Table S10: Sample size, performance, and significance for exploratory connectome-based predictive modelling (CPM) models using the 328-region parcellation. pos, positive feature model; neg, negative feature model; FC, functional coupling.

		Baseline FC				3-month ΔFC			
**Functioning**		n	r_{mean}	p	p_{FWE}	n	r_{mean}	p	p_{FWE}
6-month	*neg*	49	-0.05	.47	>.99	37	-0.06	.38	>.99
	pos		0.12	.13	.84		0.18	.07	.62
12-month	*neg*	49	-0.15	.65	>.99	36	-0.06	.45	>.99
	pos		-0.19	.77	>.99		-0.28	.89	>.99
**Symptoms**		n	r_{mean}	p	p_{FWE}	n	r_{mean}	p	p_{FWE}
6-month	*neg*	45	0.17	.07	.64	36	-0.06	.38	>.99
	pos		-0.04	.40	>.99		-0.01	.31	>.99
12-month	*neg*	45	-0.25	.86	>.99	35	-0.24	.78	>.99
	pos		-0.27	.87	>.99		-0.26	.81	>.99

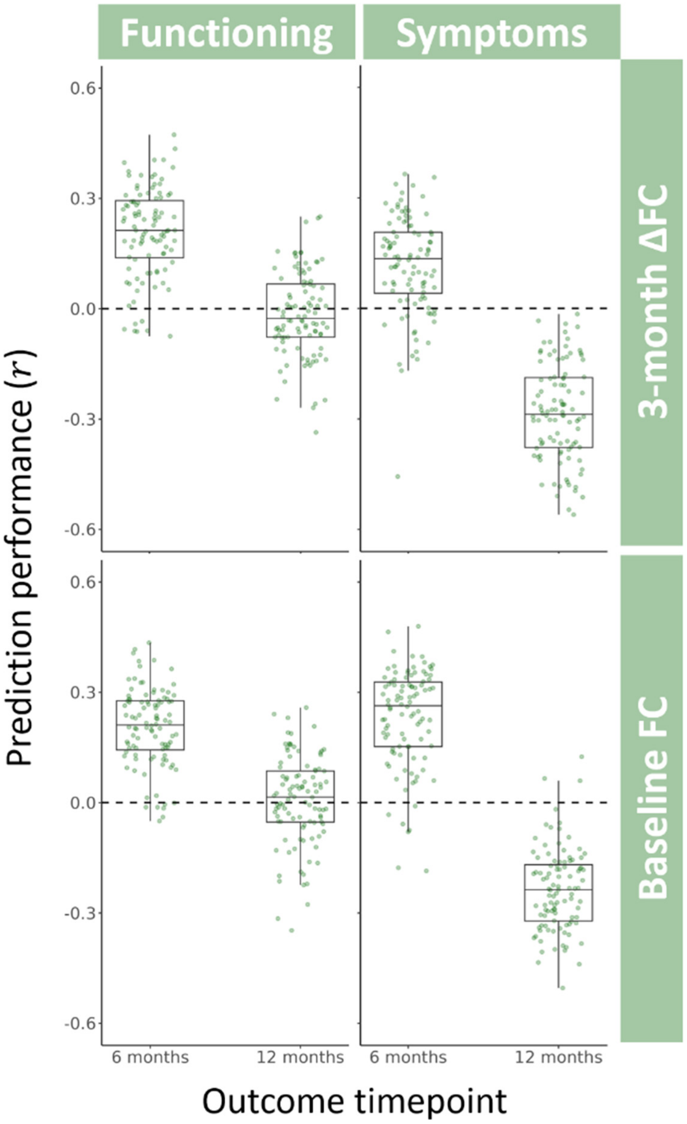

Figure S19: Performance of kernel ridge regression (KRR) for predicting patients' clinical outcomes, using the 328-region parcellation. Each data point shows the strength of Pearson's correlation between predicted and observed clinical outcomes for a single split of 4-fold cross-validation, with each of the 8 models comprising 100 random splits. FC, functional coupling.

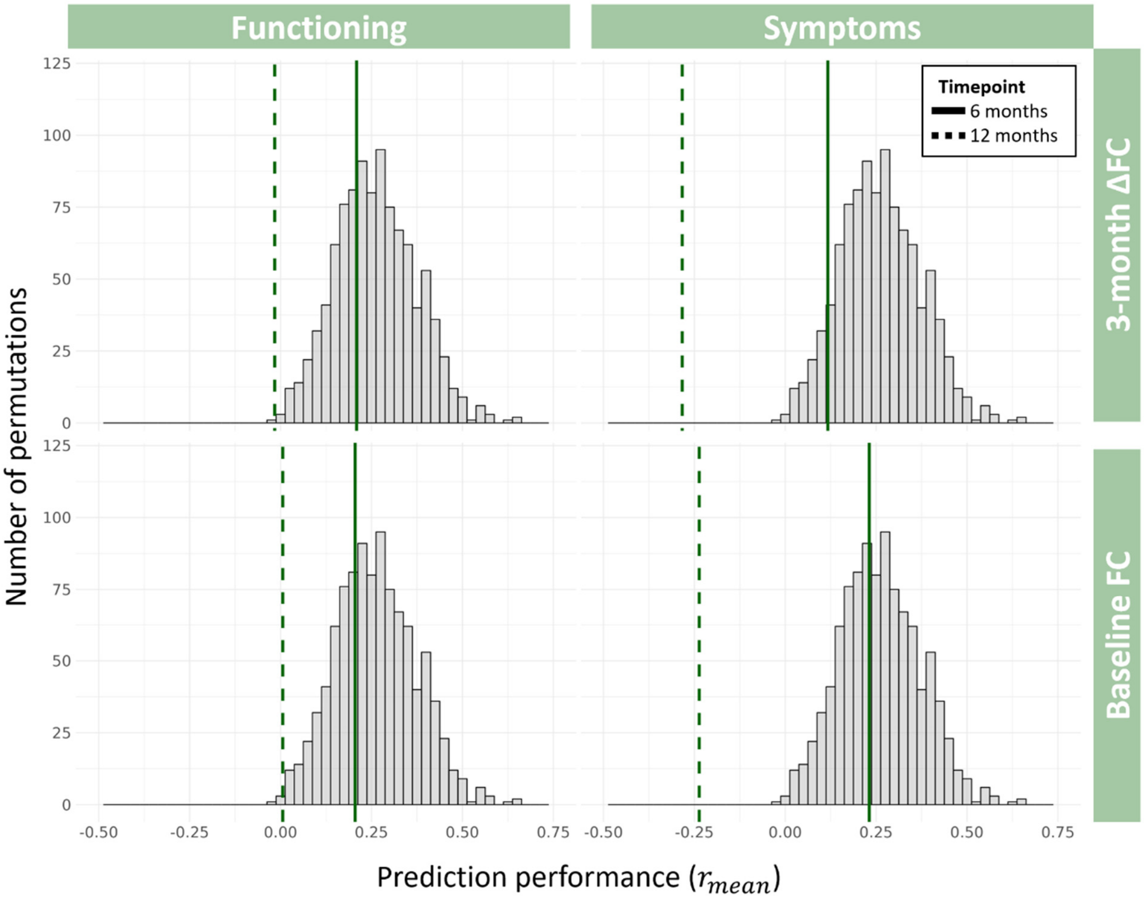

Figure S20: Prediction performance (r_{mean} shown in green) for all eight kernel ridge regression (KRR) models, using the 328-region parcellation. Model performances are superimposed against a family-wise error (FWE)-corrected empirical null distribution derived by randomly permuting clinical outcomes amongst patients. For each of 1000 permutations, 50 splits of KRR were run to calculate a single null r_{mean}. FWE correction was applied across the 8 models shown. FC, functional coupling.

Table S11: Sample size, performance, and sample size for exploratory kernel ridge regression (KRR) using the 328-region parcellation. FC, functional coupling.

	**Baseline FC**				**3-month ΔFC**			
**Functioning**	n	r_{mean}	p	p_{FWE}	n	r_{mean}	p	p_{FWE}
6-month	49	0.21	.12	.68	37	0.21	.17	.67
12-month	49	-0.01	.50	>.99	36	-0.02	.52	>.99
**Symptoms**	n	r_{mean}	p	p_{FWE}	n	r_{mean}	p	p_{FWE}
6-month	45	0.23	.12	.59	36	0.12	.32	.91
12-month	45	-0.24	.90	>.99	35	-0.28	.92	>.99

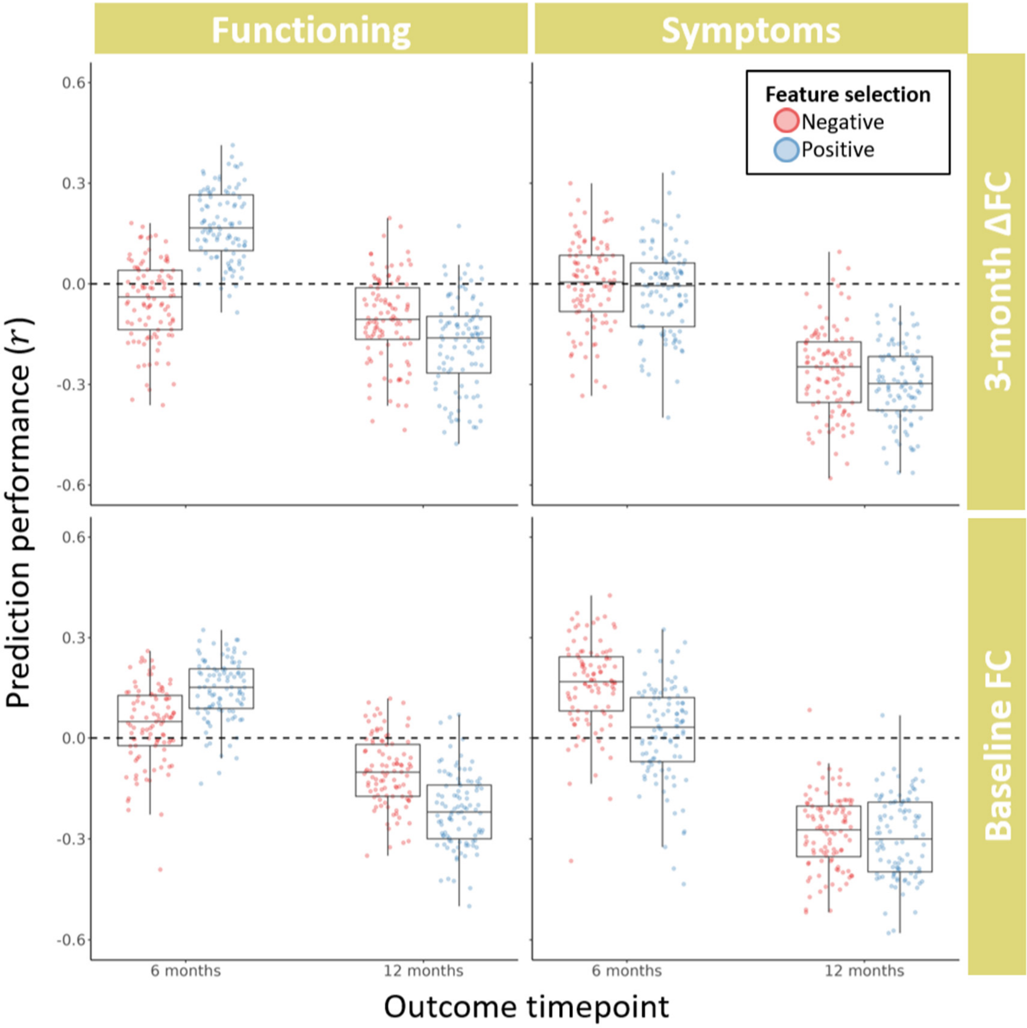

Figure S21: Performance of connectome-based predictive modelling (CPM) for predicting patients' clinical outcomes, using the 328-region parcellation and an alternate feature selection threshold of $p < .05$. Each data point shows the strength of Pearson's correlation between predicted and observed clinical outcomes for a single split of 4-fold cross-validation, with each of the 16 models comprising 100 random splits. FC, functional coupling.

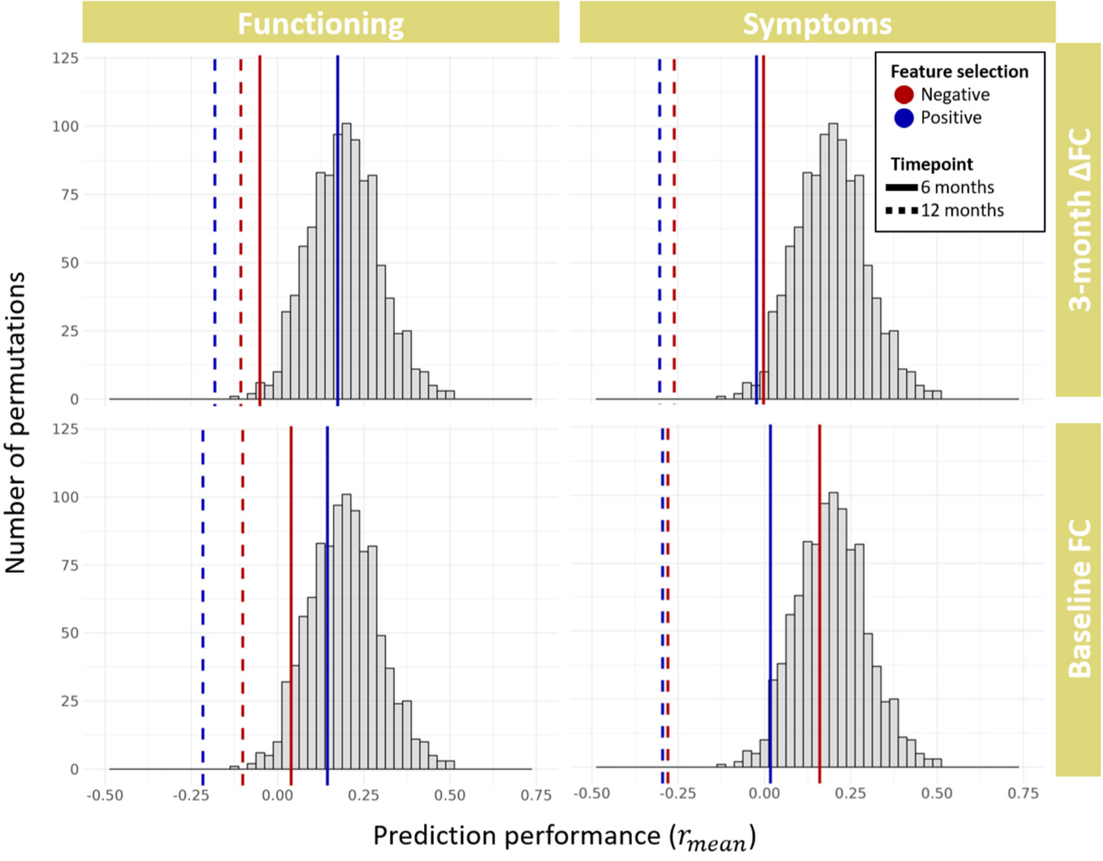

Figure S22: Prediction performance (r_{mean} shown in red and blue) for all 16 connectome-based predictive modelling (CPM) models, using the 328-region parcellation and an alternate feature selection threshold of $p < .05$. Model performances are superimposed against a family-wise error (FWE)-corrected empirical null distribution derived by randomly permuting clinical outcomes amongst patients. For each of 1000 permutations, 100 splits of CPM were run to calculate a single null r_{mean}. FWE correction was applied across the 16 models shown. FC, functional coupling.

Table S12: Sample size, performance, and significance for exploratory connectome-based predictive modelling (CPM) models using the 328-region parcellation and an alternate feature selection threshold of $p < .05$. pos, positive feature model; neg, negative feature model; FC, functional coupling.

		Baseline FC				3-month ΔFC			
**Functioning**		n	r_{mean}	p	p_{FWE}	n	r_{mean}	p	p_{FWE}
6-month	*neg*	49	0.04	.26	.94	37	-0.05	.36	.99
	pos		0.14	.08	.68		0.17	.06	.57
12-month	*neg*	49	-0.10	.52	>.99	36	-0.11	.48	>.99
	pos		-0.22	.79	>.99		-0.18	.64	>.99
**Symptoms**		n	r_{mean}	p	p_{FWE}	n	r_{mean}	p	p_{FWE}
6-month	*neg*	45	0.16	.08	.64	36	0.00	.27	.98
	pos		0.02	.27	.97		-0.02	.31	.99
12-month	*neg*	45	-0.28	.88	>.99	35	-0.26	.80	>.99
	pos		-0.30	.89	>.99		-0.30	.86	>.99

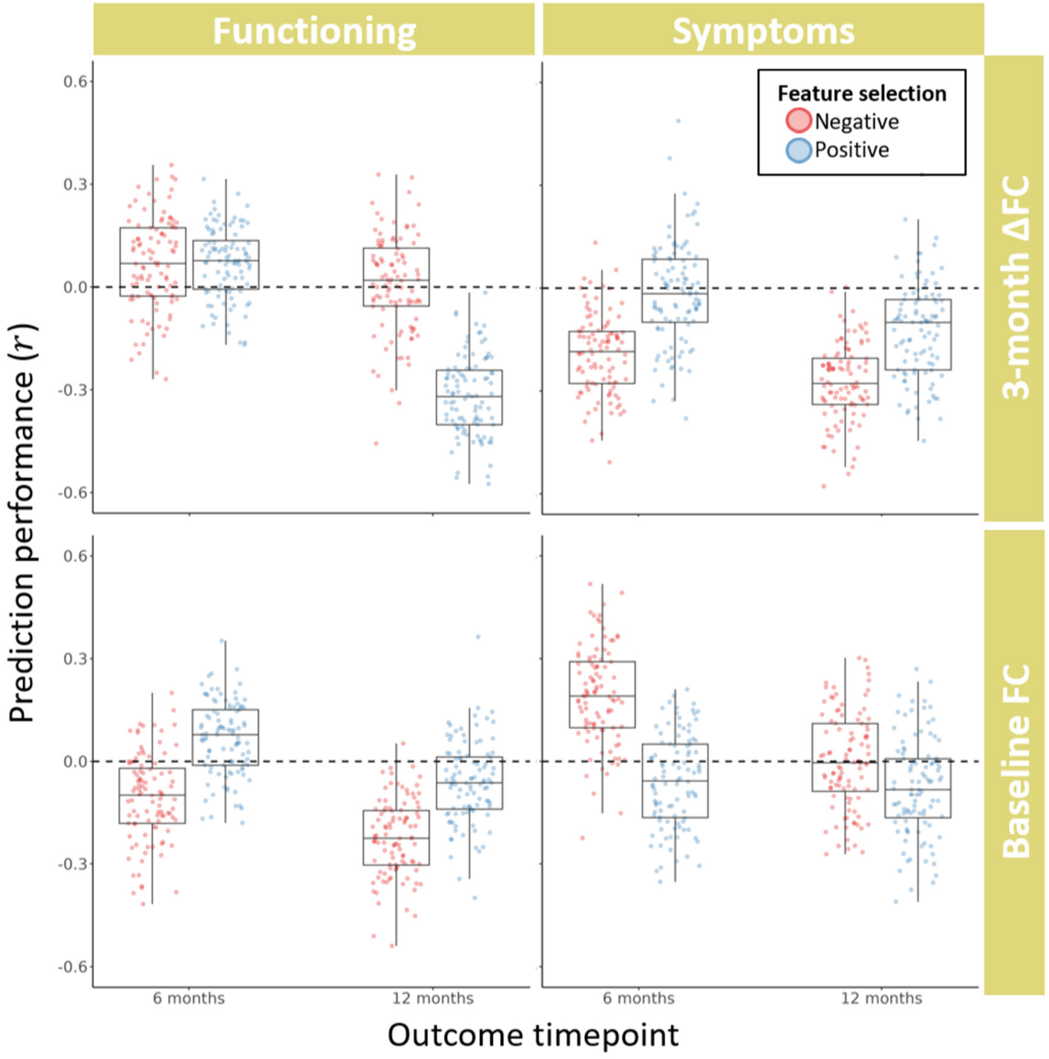

Figure S23: Performance of connectome-based predictive modelling (CPM) for predicting patients' clinical outcomes, using the 328-region parcellation and an alternate feature selection threshold of $p < .001$. Each data point shows the strength of Pearson's correlation between predicted and observed clinical outcomes for a single split of 4-fold cross-validation, with each of the 16 models comprising 100 random splits. FC, functional coupling.

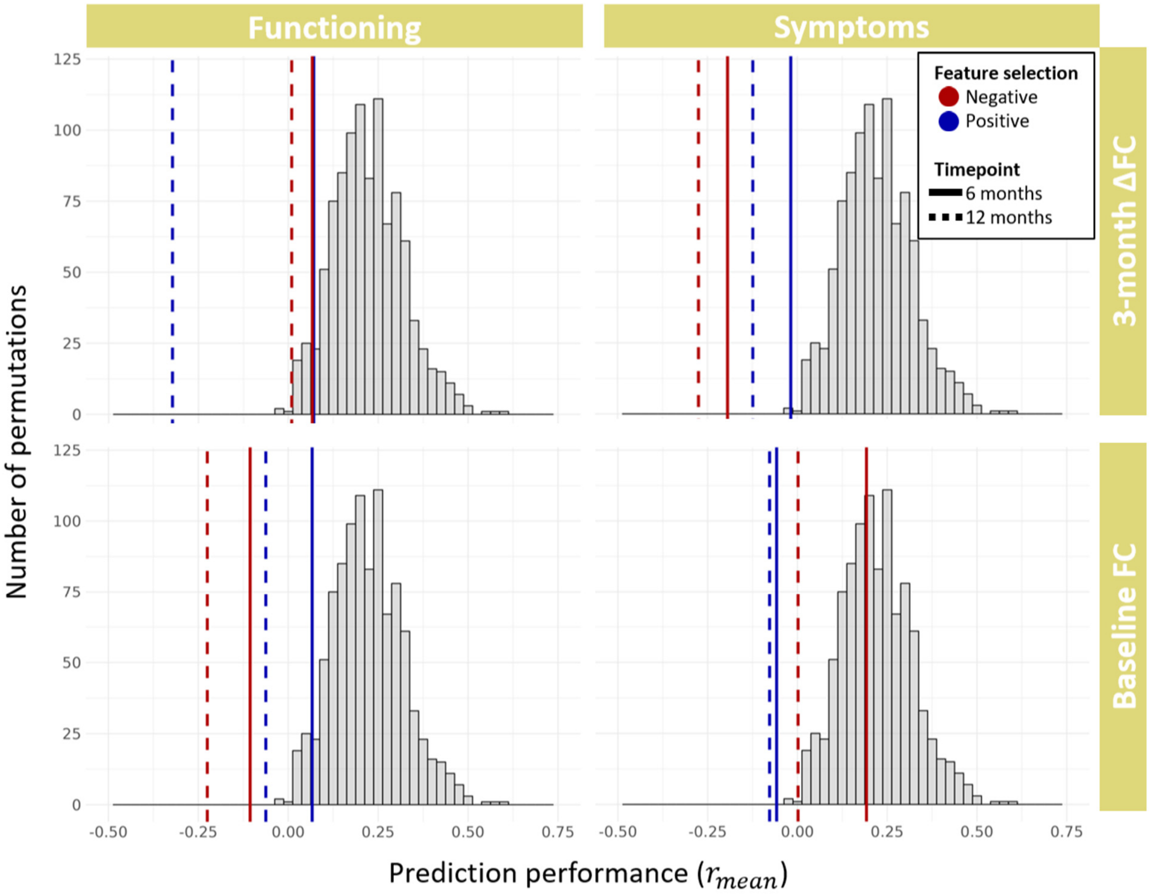

Figure S24: Prediction performance (r_{mean} shown in red and blue) for all 16 connectome-based predictive modelling (CPM) models, using the 328-region parcellation and an alternate feature selection threshold of $p < .001$. Model performances are superimposed against a family-wise error (FWE)-corrected empirical null distribution derived by randomly permuting clinical outcomes amongst patients. For each of 1000 permutations, 100 splits of CPM were run to calculate a single null r_{mean}. FWE correction was applied across the 16 models shown. FC, functional coupling.

Table S13: Sample size, performance, and significance for exploratory connectome-based predictive modelling (CPM) models using the 328-region parcellation and an alternate feature selection threshold of $p < .001$. pos, positive feature model; neg, negative feature model; FC, functional coupling.

		Baseline FC				3-month ΔFC			
**Functioning**		n	r_{mean}	p	p_{FWE}	n	r_{mean}	p	p_{FWE}
6-month	*neg*	49	-0.11	.67	>.99	37	0.07	.20	.95
	pos		0.07	.20	.95		0.07	.19	.95
12-month	*neg*	49	-0.23	.91	>.99	36	0.01	.31	>.99
	pos		-0.06	.51	>.99		-0.32	.97	>.99
**Symptoms**		n	r_{mean}	p	p_{FWE}	n	r_{mean}	p	p_{FWE}
6-month	*neg*	45	0.19	.06	.60	36	-0.19	.80	>.99
	pos		-0.06	.50	>.99		-0.02	.34	>.99
12-month	*neg*	45	0.00	.33	>.99	35	-0.27	.94	>.99
	pos		-0.08	.53	>.99		-0.12	.63	>.99

References

1. Orygen Youth Health., Early Psychosis Prevention & Intervention Centre (Vic.). Cognitive-behavioural case management in early psychosis : a handbook. Parkville, Vic: Orygen Youth Health; 2010.

2. O'Donoghue B, Francey SM, Nelson B, Ratheesh A, Allott K, Graham J, et al. Staged treatment and acceptability guidelines in early psychosis study (STAGES): A randomized placebo controlled trial of intensive psychosocial treatment plus or minus antipsychotic medication for first-episode psychosis with low-risk of self-harm or aggression. Study protocol and baseline characteristics of participants. Early Intervention in Psychiatry. 2019;13(4):953–60.

3. Francey SM, O'Donoghue B, Nelson B, Graham J, Baldwin L, Yuen HP, et al. Psychosocial Intervention With or Without Antipsychotic Medication for First-Episode Psychosis: A Randomized Noninferiority Clinical Trial. Schizophrenia Bulletin Open. 2020 Jan 1;1(1):sgaa015.

4. Esteban O, Birman D, Schaer M, Koyejo OO, Poldrack RA, Gorgolewski KJ. MRIQC: Advancing the automatic prediction of image quality in MRI from unseen sites. PLOS ONE. 2017 Sep 25;12(9):e0184661.

5. Esteban O, Markiewicz CJ, Blair RW, Moodie CA, Isik AI, Erramuzpe A, et al. fMRIPrep: a robust preprocessing pipeline for functional MRI. Nat Methods. 2019 Jan;16(1):111–6.

6. Tustison NJ, Avants BB, Cook PA, Zheng Y, Egan A, Yushkevich PA, et al. N4ITK: Improved N3 Bias Correction. IEEE Transactions on Medical Imaging. 2010 Jun;29(6):1310–20.

7. Avants BB, Epstein CL, Grossman M, Gee JC. Symmetric diffeomorphic image registration with cross-correlation: Evaluating automated labeling of elderly and neurodegenerative brain. Medical Image Analysis. 2008 Feb 1;12(1):26–41.

8. Zhang Y, Brady M, Smith S. Segmentation of brain MR images through a hidden Markov random field model and the expectation-maximization algorithm. IEEE Transactions on Medical Imaging. 2001 Jan;20(1):45–57.

9. Dale AM, Fischl B, Sereno MI. Cortical Surface-Based Analysis: I. Segmentation and Surface Reconstruction. NeuroImage. 1999 Feb 1;9(2):179–94.

10. Fonov V, Evans A, McKinstry R, Almli C, Collins D. Unbiased nonlinear average age-appropriate brain templates from birth to adulthood. NeuroImage. 2009 Jul 1;47:S102.

11. Jenkinson M, Bannister P, Brady M, Smith S. Improved optimization for the robust and accurate linear registration and motion correction of brain images. Neuroimage. 2002 Oct;17(2):825–41.

12. Cox RW. AFNI: Software for Analysis and Visualization of Functional Magnetic Resonance Neuroimages. Computers and Biomedical Research. 1996 Jun 1;29(3):162–73.

13. Sladky R, Friston KJ, Tröstl J, Cunnington R, Moser E, Windischberger C. Slice-timing effects and their correction in functional MRI. NeuroImage. 2011 Sep 15;58(2):588–94.

14. Hutton C, Bork A, Josephs O, Deichmann R, Ashburner J, Turner R. Image Distortion Correction in fMRI: A Quantitative Evaluation. NeuroImage. 2002 May 1;16(1):217–40.

15. Friston KJ, Williams S, Howard R, Frackowiak RSJ, Turner R. Movement-Related effects in fMRI time-series. Magnetic Resonance in Medicine. 1996;35(3):346–55.

16. Van Dijk KRA, Sabuncu MR, Buckner RL. The influence of head motion on intrinsic functional connectivity MRI. NeuroImage. 2012 Jan 2;59(1):431–8.

17. Power JD, Barnes KA, Snyder AZ, Schlaggar BL, Petersen SE. Spurious but systematic correlations in functional connectivity MRI networks arise from subject motion. NeuroImage. 2012 Feb 1;59(3):2142–54.

18. Parkes L, Fulcher B, Yücel M, Fornito A. An evaluation of the efficacy, reliability, and sensitivity of motion correction strategies for resting-state functional MRI. NeuroImage. 2018 May 1;171:415–36.

19. Pruim RHR, Mennes M, van Rooij D, Llera A, Buitelaar JK, Beckmann CF. ICA-AROMA: A robust ICA-based strategy for removing motion artifacts from fMRI data. NeuroImage. 2015 May 15;112:267–77.

20. Chen Z, Calhoun V. Effect of Spatial Smoothing on Task fMRI ICA and Functional Connectivity. Front Neurosci. 2018 Feb 2;12:15.

21. Power JD, Plitt M, Laumann TO, Martin A. Sources and implications of whole-brain fMRI signals in humans. NeuroImage. 2017 Feb 1;146:609–25.

22. Murphy K, Fox MD. Towards a consensus regarding global signal regression for resting state functional connectivity MRI. NeuroImage. 2017 Jul 1;154:169–73.

23. Birn RM, Diamond JB, Smith MA, Bandettini PA. Separating respiratory-variation-related fluctuations from neuronal-activity-related fluctuations in fMRI. Neuroimage. 2006 Jul 15;31(4):1536–48.

24. Liu TT, Nalci A, Falahpour M. The global signal in fMRI: Nuisance or Information? Neuroimage. 2017 Apr 15;150:213–29.

25. Turchi J, Chang C, Ye FQ, Russ BE, Yu DK, Cortes CR, et al. The Basal Forebrain Regulates Global Resting-State fMRI Fluctuations. Neuron. 2018 Feb 21;97(4):940-952.e4.

26. Schölvinck ML, Maier A, Ye FQ, Duyn JH, Leopold DA. Neural basis of global resting-state fMRI activity. Proceedings of the National Academy of Sciences. 2010 Jun;107(22):10238–43.

27. Li J, Kong R, Liégeois R, Orban C, Tan Y, Sun N, et al. Global signal regression strengthens association between resting-state functional connectivity and behavior. NeuroImage. 2019 Aug 1;196:126–41.

28. Aquino KM, Fulcher BD, Parkes L, Sabaroedin K, Fornito A. Identifying and removing widespread signal deflections from fMRI data: Rethinking the global signal regression problem. NeuroImage. 2020 May 15;212:116614.

29. Satterthwaite TD, Elliott MA, Gerraty RT, Ruparel K, Loughead J, Calkins ME, et al. An improved framework for confound regression and filtering for control of motion artifact in the preprocessing of resting-state functional connectivity data. Neuroimage. 2013 Jan 1;64:240–56.

30. Lindquist MA, Geuter S, Wager TD, Caffo BS. Modular preprocessing pipelines can reintroduce artifacts into fMRI data. Hum Brain Mapp. 2019 Jan 21;40(8):2358–76.

31. Schaefer A, Kong R, Gordon EM, Laumann TO, Zuo XN, Holmes AJ, et al. Local-Global Parcellation of the Human Cerebral Cortex from Intrinsic Functional Connectivity MRI. Cereb Cortex. 2018 Sep 1;28(9):3095–114.

32. Fischl B, Salat DH, Busa E, Albert M, Dieterich M, Haselgrove C, et al. Whole Brain Segmentation: Automated Labeling of Neuroanatomical Structures in the Human Brain. Neuron. 2002 Jan 31;33(3):341–55.

33. Fischl B, Salat DH, van der Kouwe AJW, Makris N, Ségonne F, Quinn BT, et al. Sequence-independent segmentation of magnetic resonance images. NeuroImage. 2004 Jan 1;23:S69–84.

34. Tian Y, Margulies DS, Breakspear M, Zalesky A. Topographic organization of the human subcortex unveiled with functional connectivity gradients. Nat Neurosci. 2020 Nov;23(11):1421–32.

35. Brown JA, Deng J, Neuhaus J, Sible IJ, Sias AC, Lee SE, et al. Patient-Tailored, Connectivity-Based Forecasts of Spreading Brain Atrophy. Neuron. 2019 Dec 4;104(5):856-868.e5.
